# Orange Juice as a Substrate for Milk and Water Kefir Grains: Production and Characterization of Novel Kefir-like Beverages

**DOI:** 10.3390/foods15193544

**Published:** 2026-10-04

**Authors:** Eduardo Balvis Outeiriño, Marta Abajo Justel, Nelson Pérez Guerra

**Affiliations:** 1 Department of Systems Engineering and Automation, Faculty of Sciences, University of Vigo, Ourense Campus, As Lagoas s/n, 32004 Ourense, Spain; ebalvis@uvigo.gal; 2 Industrial Biotechnology and Environmental Engineering Group “BiotecnIA”, Department of Analytical and Food Chemistry, University of Vigo, Ourense Campus, As Lagoas s/n, 32004 Ourense, Spain; venexoc@gmail.com

**Keywords:** kefir-like beverages, organic acids, alcohols, vitamins content, mineral composition, lactic acid bacteria, acetic acid bacteria, yeasts

## Abstract

Orange juice was investigated as a substrate for the production of novel kefir-like beverages using milk kefir grains (MKG) and water kefir grains (WKG). Fermentation was monitored for 48 h to characterize changes in physicochemical parameters, substrate consumption, metabolite production, microbial populations, antibacterial activity, vitamin and mineral composition, and volatile compounds. Both kefir grain types supported the growth of lactic acid bacteria, acetic acid bacteria, and yeasts, reaching final populations of approximately 6–7, 5–6, and 8 log CFU/mL, respectively. WKG promoted greater early production of lactic acid and consistently higher levels of acetic acid, ethanol, and antibacterial activity, whereas MKG produced a slightly higher lactic acid concentration after 48 h and was associated with greater increases in potassium, phosphorus, magnesium, and calcium. Vitamins B1, B2, and C decreased during fermentation with both MKG and WKG. The volatile profile was substantially altered during fermentation, with the potential aroma contribution shifting from predominantly balsamic and citrus notes toward fruity and sweet characteristics, particularly after 48 h. Overall, kefir grain type and fermentation time strongly influenced the physicochemical, microbiological, nutritional, antibacterial, and volatile characteristics of fermented orange juice. These findings demonstrate the potential of both grains for developing non-dairy kefir-like beverages.

## 1. Introduction

Kefir is a fermented beverage traditionally produced by inoculating milk with kefir grains, complex symbiotic consortia of lactic acid bacteria (LAB), acetic acid bacteria (AAB), and yeasts embedded in an extracellular polysaccharide matrix known as kefiran [1,2,3]. During fermentation, these microorganisms convert carbohydrates into organic acids, ethanol, carbon dioxide, glycerol, vitamins, exopolysaccharides, and numerous bioactive metabolites that contribute to the nutritional, sensory, and functional properties of kefir [2,4,5]. Owing to its rich microbial diversity and the presence of microorganisms with probiotic potential, kefir has attracted considerable attention as a functional food associated with antimicrobial, antioxidant, anti-inflammatory, immunomodulatory, and gastrointestinal health-promoting effects [3,5,6].

In recent years, the growing consumer demand for plant-based and lactose-free functional foods has stimulated the development of non-dairy kefir-like beverages produced from fruit juices, vegetables, cereals, and other sugar-rich substrates [6,7,8,9]. Fruit juices are particularly attractive substrates because they naturally contain fermentable sugars, organic acids, vitamins, minerals, and phytochemicals that support microbial growth while preserving desirable sensory characteristics [8,9]. Among them, orange juice is one of the most widely consumed fruit beverages worldwide and represents an excellent source of vitamin C, folates, carotenoids, flavonoids, potassium, and other micronutrients associated with antioxidant activity and a reduced risk of chronic diseases [10,11].

The production of non-dairy kefir-like beverages offers several advantages. Compared with traditional milk kefir, these products may contain higher levels of dietary fiber and unsaturated fatty acids and exhibit greater antioxidant capacity [12]. In addition, they are suitable for consumers who are lactose intolerant, allergic to milk proteins, have hypercholesterolemia, or follow vegan or vegetarian diets [6,13,14,15,16]. Furthermore, fruit-based kefir-like beverages could provide an additional outlet for fruit with limited market value [17,18], although this aspect was not specifically addressed in the present study, which used commercial fresh orange juice.

The microbial metabolism associated with kefir fermentation substantially modifies the physicochemical and nutritional characteristics of fruit juices. Carbohydrate metabolism results in the production of lactic acid, acetic acid, ethanol, glycerol, and other metabolites that influence acidity, flavor, microbial stability, and functional properties [7,19]. At the same time, the microbial consortium may synthesize or consume vitamins and alter mineral bioavailability through the enzymatic degradation of mineral-binding compounds or the release of intracellular constituents during fermentation [20,21,22]. The extent of these changes depends on several factors, including substrate composition, fermentation conditions, and, importantly, the microbial composition of the kefir grains [2,7].

Milk kefir grains (MKG) and water kefir grains (WKG) differ considerably in their microbial ecology and metabolic capabilities. MKG are typically dominated by species of *Lactobacillus*, *Lactococcus*, *Leuconostoc*, *Acetobacter*, and various yeasts, whereas WKG harbor a distinct consortium adapted to sugar-rich environments, commonly including species of *Lactobacillus*, *Leuconostoc*, *Bifidobacterium*, *Acetobacter*, *Saccharomyces*, *Hanseniaspora*, and *Dekkera* [18]. Consequently, beverages produced with these two types of kefir grains often differ in organic acid production, alcohol formation, microbial growth, and sensory characteristics [6,7,18,23].

Previous studies on fruit-based kefir beverages have focused primarily on microbial viability, physicochemical characteristics, antioxidant activity, phenolic compounds, and volatile profiles [6,8,9,24]. In contrast, limited information is available regarding the effects of kefir fermentation on vitamin composition, particularly the simultaneous changes in B-group vitamins, vitamin C, and vitamin D, as well as on mineral composition during the fermentation of non-dairy substrates. Moreover, comparative studies investigating the fermentation dynamics of orange juice using milk and water kefir grains under identical conditions are still limited.

Therefore, the aim of this study was to compare the fermentation of orange juice using milk kefir grains and water kefir grains by evaluating: (i) the time-course changes in culture pH, sugars, proteins, organic acids, ethanol, glycerol, biomass, and antibacterial activity; (ii) the dynamics of lactic acid bacteria, acetic acid bacteria, yeasts, *Pseudomonas* spp., and Enterobacteriaceae throughout fermentation and; (iii) changes in vitamin and mineral composition; and (iv) alterations in volatile compounds. A better understanding of these differences will contribute to optimizing fruit-based kefir fermentations and support the development of non-dairy fermented beverages.

## 2. Materials and Methods

### 2.1. Inoculum Preparation

Milk kefir grains (MKG) and water kefir grains (WKG) were purchased from Kefiralia (Burumart Commerce S.L., Arrasate, Guipúzcoa, Spain) in sealed plastic zipper bags. Both types of kefir grains were stored at 4 °C until activation. The methods used to determine the viable counts of lactic acid bacteria (LAB), acetic acid bacteria (AAB), yeasts, *Pseudomonas* spp., and Enterobacteriaceae in MKG and WKG have been previously reported [23]. The initial viable counts in the activated grains used for inoculation were: MKG, LAB, 7.96 ± 0.03; AAB, 6.81 ± 0.06; and yeasts, 7.93 ± 0.04 log CFU/g; and WKG, LAB, 6.82 ± 0.05; AAB, 6.78 ± 0.03; and yeasts, 7.25 ± 0.05 log CFU/g [23]. *Pseudomonas* spp. and Enterobacteriaceae were below 1.48 log CFU/g in both grain types.

**Figure 1:**
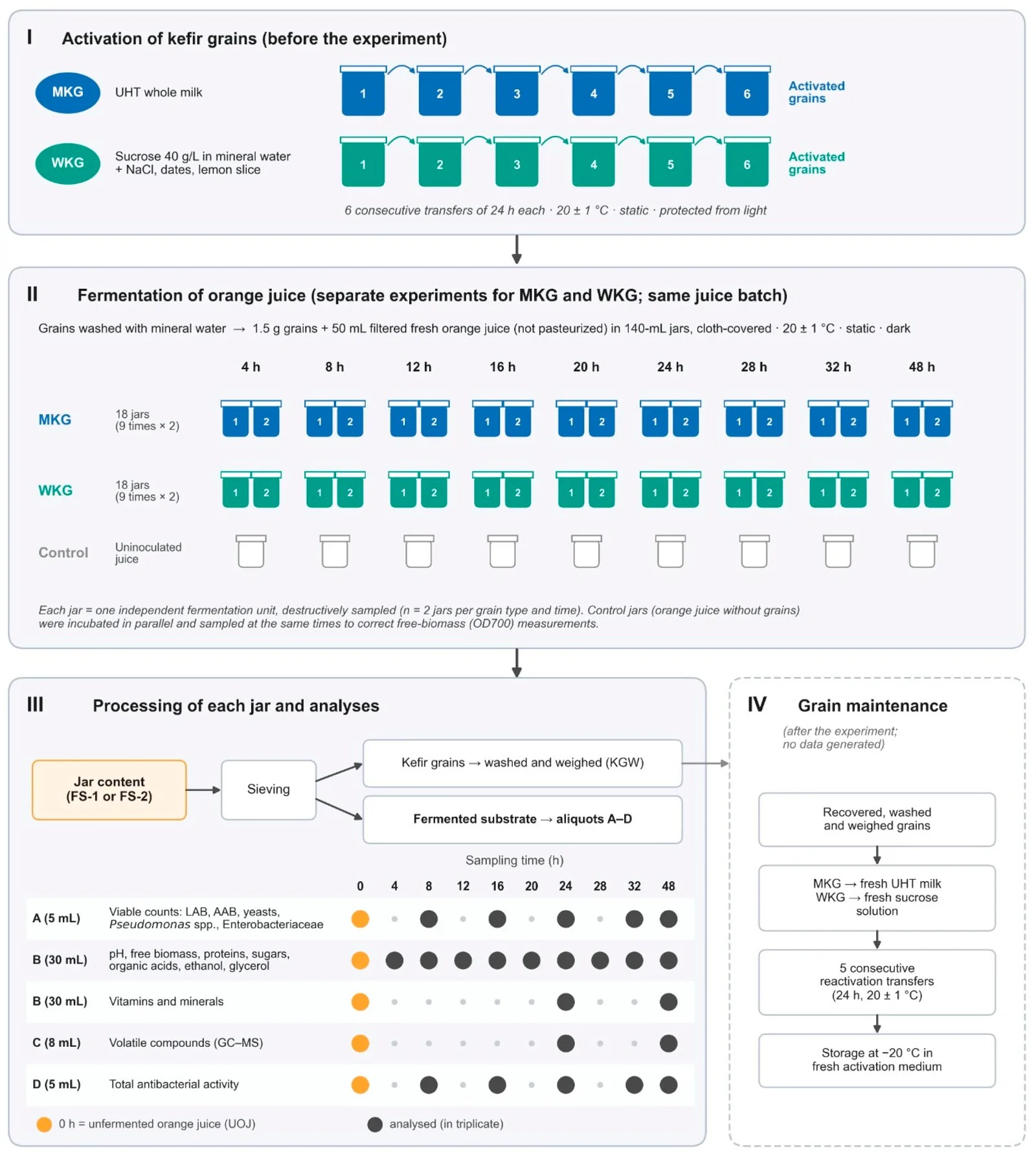
Experimental design. (**I**) Activation of milk kefir grains (MKG) and water kefir grains (WKG) in their respective activation media. (**II**) Fermentation of orange juice: for each grain type, 18 jars (two independent jars for each of nine sampling times) were sampled; uninoculated juice jars were incubated in parallel as controls for free-biomass correction. (**III**) Processing of each jar and analyses performed at each sampling time. (**IV**) Post-experiment maintenance of the recovered grains, which did not generate data.

MKG were activated in ultra-high-temperature (UHT) whole milk with the following mean composition: lactose, 46.84 ± 2.18 g/L; proteins, 31.99 ± 0.06 g/L; total nitrogen, 5.40 ± 0.01 g/L; and pH, 6.70 ± 0.03, as described by Bazán et al. [25]. WKG were activated in a sucrose solution (40 g/L in mineral water) supplemented with a small amount of sodium chloride (NaCl), two dates, and one slice of lemon, following the procedure described by Balvis et al. [23]. The activation procedure consisted of six consecutive cycles of 24 h each at room temperature (20 ± 1 °C), without agitation and protected from direct sunlight (Figure 1).

UHT whole milk (Central Lechera Asturiana, Asturias, Spain), freshly squeezed orange juice (Hacendado, Mercadona, Spain), sucrose (Hacendado, Mercadona, Spain), mineral water (Cabreiroá, Ourense, Spain), salt (Hacendado, Mercadona, Spain), dates (Hacendado, Mercadona, Spain), and lemons (Frutas Poveda S.A., Murcia, Spain) were purchased from a local Mercadona supermarket (Mercadona S.A., Ourense, Spain).

### 2.2. Fermentation Conditions and Sampling

For the production of kefir-like beverages, fresh, not-from-concentrate orange juice (100% orange juice, with no added sugar, preservatives, or other ingredients; Hacendado, Mercadona, Spain) was used as the fermentation substrate. The juice was freshly squeezed in-store immediately before purchase from Valencia Late oranges (*Citrus sinensis*) originating from Argentina. The juice was neither pasteurized nor refrigerated prior to use. No additional thermal treatment was applied before fermentation, as heat treatment was deliberately avoided to minimize potential losses of heat-sensitive compounds, particularly vitamins. The juice has the following composition: pH 3.97 ± 0.00; glucose, 36.62 ± 0.03 g/L; fructose, 36.62 ± 0.43 g/L; sucrose, 43.49 ± 0.04 g/L; citric acid, 6.88 ± 0.11 g/L; malic acid, 2.98 ± 0.05 g/L; succinic acid, 0.33 ± 0.03 g/L; total protein, 6.00 ± 0.11 g/L; and ascorbic acid, 405.35 ± 0.01 mg/L.

Prior to inoculation, the juice was filtered through a sterile cloth to remove suspended solids. Subsequently, 50 mL of filtered orange juice was inoculated with 1.5 g of the corresponding kefir grains, which had been previously separated from the activation medium by sieving and washed with mineral water (Cabreiroá, Ourense, Spain) to remove the adhering medium.

Fermentations were performed separately using milk kefir grains (MKG) and water kefir grains (WKG), with the same batch of orange juice used for both fermentation experiments (Figure 1). For each kefir-grain type, 18 sterilized 140-mL glass jars were used (36 inoculated jars in total), with two jars assigned to each of the nine sampling times (4, 8, 12, 16, 20, 24, 28, 32, and 48 h). Each jar was an independent fermentation unit that was sampled at its assigned time; thus, two independent fermentation jars were obtained for each kefir-grain type and sampling time within a single fermentation run. During incubation, the jars were covered with sterile breathable cloth secured with a rubber band and maintained at room temperature (20 ± 1 °C), without agitation and protected from direct sunlight [23].

At each sampling time, the fermented substrates obtained from the two independent jars were processed separately. The fermented substrates were filtered through a plastic sieve to separate the kefir grains, and the resulting samples were designated FS-1 and FS-2. Each sample was divided into four aliquots: aliquot A (5 mL) for microbiological analysis and viable-cell enumeration; aliquot B (30 mL) for determination of free biomass, proteins, sugars, organic acids, alcohols, vitamins, and minerals; aliquot C (8 mL) for volatile compound analysis; and aliquot D (5 mL) for determination of total antibacterial activity. Unless otherwise specified, all analytical determinations were performed in triplicate.

The kefir grains recovered after fermentation were washed with mineral water (Cabreiroá, Ourense, Spain) to remove residual orange juice and subsequently weighed to determine their wet weight. MKG were then transferred to fresh UHT whole milk, whereas WKG were transferred to the sucrose solution described in Section 2.1. These grains were not used for further fermentations of orange juice; they were maintained by five consecutive 24-h transfers in their original activation medium at room temperature (20 ± 1 °C), without agitation and protected from direct sunlight, and then stored at −20 °C in fresh activation medium for future use (Figure 1). These maintenance transfers did not generate any of the data reported in this study.

### 2.3. Quantification of Viable Cell Counts

The viable populations of lactic acid bacteria (LAB), acetic acid bacteria (AAB), yeasts, Enterobacteriaceae, and *Pseudomonas* spp. were determined in unfermented orange juice and in fermented substrates collected after 8, 16, 24, 32, and 48 h of fermentation. Enterobacteriaceae, and *Pseudomonas* spp. were also enumerated in both kefir and milk kefir grains. The analytical procedure was based on previously described methods [23,25].

Briefly, samples were serially diluted as appropriate, and aliquots of the corresponding dilutions were inoculated in triplicate onto the corresponding selective culture media for the enumeration of each microbial group: LAB, on MRS agar containing amphotericin B (0.1 g/L); AAB, on Carr agar containing amphotericin B (0.1 g/L); yeasts, on yeast extract–glucose agar containing chloramphenicol (0.1 g/L); Enterobacteriaceae, on double-layered violet red bile glucose agar; and *Pseudomonas* spp., on *Pseudomonas* agar base (PAB) containing cetrimide–fucidin (10 g/L) [23,25].

The incubation conditions were as follows: LAB were incubated at 30 °C for 48 h under anaerobic conditions, whereas AAB were incubated at 30 °C for 48 h, yeasts at 25 °C for 48 h, Enterobacteriaceae at 37 °C for 24 h, and *Pseudomonas* spp. at 20 °C for 48 h, all under aerobic conditions.

Following incubation, colonies were counted, and viable populations were calculated using the appropriate dilution factors [23,25]. Results were expressed as logarithms of colony-forming units per milliliter (log CFU/mL).

### 2.4. Quantification of Free Biomass

To quantify the free biomass concentration in unfermented and fermented orange juice, aliquot B was centrifuged at 5000 rpm for 5 min at 4 °C. The resulting pellets were washed twice with 0.8% (*w*/*v*) NaCl, with centrifugation performed under the same conditions after each washing step. The washed pellets were then resuspended in the same saline solution, and the optical density of the suspensions was measured at 700 nm [25]. Finally, the optical density values were converted into free biomass concentrations (g/L) using a previously established calibration curve relating cell dry weight to optical density.

To account for potential interference caused by suspended solids and naturally occurring colored compounds in orange juice [26], as well as by the growth of the indigenous microbiota, control samples consisting of orange juice without kefir grains were incubated under the same conditions as the fermented samples. These control samples were collected at the same sampling times as the MKG- and WKG-fermented samples and were used to correct the biomass measurements.

### 2.5. Chemical Composition of the Substrate and Fermented Beverages

The concentrations of sugars, organic acids, alcohols, proteins, vitamins, and minerals were determined in unfermented orange juice and in the fermented beverages. Sugars, organic acids, and alcohols were quantified at all sampling times, whereas vitamins and minerals were determined in unfermented orange juice and after 24 and 48 h of fermentation.

For determination of sugars, organic acids, and alcohols, samples were filtered through hydrophilic polytetrafluoroethylene (PTFE) syringe filters (0.2 µm pore size) before HPLC analysis. Glucose, fructose, sucrose, ethanol, glycerol, lactic acid, acetic acid, malic acid, citric acid, and succinic acid were quantified using an Agilent 1200 HPLC system (Agilent, Waldbronn, Germany) equipped with an ION-300 Organic Acids column (300 mm × 7.8 mm internal diameter) and an IONGUARD™ polymeric guard column (both obtained Tecknokroma S. Coop. C. Ltd.a, Barcelona, Spain). Separation was performed at 60–65 °C using 0.012 N sulfuric acid as the mobile phase at a flow rate of 0.4 mL/min. Compounds were detected using a refractive-index detector. The injection volume was 10 µL and the run time was 40 min.

External calibration curves were prepared using analytical-grade standards (Merck, Darmstadt, Germany and Aldrich (Buchs, Switzerland)) at concentrations ranging from 0.1 to 10.0 g/L (R^2^ ≥ 0.9978), and quantification was based on peak areas. All analytical determinations were performed in triplicate [25].

Protein concentration was determined in triplicate using the Lowry method, with bovine serum albumin used to construct the calibration curve. The calibration range was 0.05–0.50 g/L (R^2^ = 0.9953). Absorbance was measured at 750 nm [23].

For mineral analysis, 2-g samples were digested in triplicate with 1 mL of hydrogen peroxide (35 wt%) and 6 mL of trace-metal-grade nitric acid. Analytical blanks were prepared using the same procedure. Microwave digestion was performed at 1000 W using a Mars 6 microwave digester, with a 15-min ramp to 195 °C, followed by a 20-min hold at 195 °C. The digested samples were subsequently diluted to 50 mL with ultrapure water. Mineral concentrations were determined using an Agilent 5800 inductively coupled plasma optical emission spectrometer (ICP-OES), with rhodium used as the internal standard. A certified multielement calibration solution was used to construct the calibration curves. The coefficient of determination (R^2^) was >0.9999 for all calibration curves, and recovery percentages ranged from 92% to 108% [23]. The emission lines used were as follows (nm): K, 766.491; Mg, 280.270; Ca, 393.366; Na, 588.995; Fe, 238.204; Zn, 213.857; Cu, 324.754; and Se, 196.026. The limits of detection (LOD) were as follows (mg/L): K, 0.09764; Mg, 0.00031; Ca, 0.00044; Na, 0.01379; Fe, 0.00018; Zn, 0.00029; Cu, 0.00052; and Se, 0.000029. The limits of quantification (LOQ) were 0.01 mg/L for Fe, Cu, Mg, Se, and Zn, 0.10 mg/L for Na and Ca, and 0.50 mg/L for K.

Phosphorus concentration was quantified as described by Gliszczyńska-Świgło and Rybicka [27], with LOD, LOQ, and R^2^ values of 0.37 µg/mL, 1.13 µg/mL, and 0.999, respectively.

For vitamin analysis, samples were mixed with 50% acetonitrile in acidified water, vigorously shaken for 1 min, and roller-mixed for 10 min while protected from light. The samples were then centrifuged at 10,000× *g* for 10 min, and the supernatants were filtered through hydrophilic PTFE syringe filters (0.2 µm). A composite vitamin standard solution was prepared daily. Thiamine (B1), riboflavin (B2), nicotinic acid (B3), pyridoxal, pyridoxine, pyridoxamine, and ascorbic acid (C) were quantified by HPLC–tandem mass spectrometry using an Agilent 1260 Series HPLC coupled to a SCIEX Triple Quad 3500 mass spectrometer equipped with an electrospray ionization source. Chromatographic separation was achieved using a Luna C18 column (150 mm × 2 mm, 3 µm). The mobile phase consisted of water containing 0.1% formic acid (A) and acetonitrile containing 0.1% formic acid (B), using the gradient described by Balvis et al. [23]. The flow rate was 400 µL/min and the column temperature was maintained at 40 °C. Nitrogen was used as both nebulizer and collision gas [23]. A composite standard solution was prepared daily by mixing and diluting individual vitamin stock solutions (Sigma-Aldrich, St. Louis, MO, USA), prepared from commercial standards (purity > 98%), with Milli-Q water to obtain a final concentration of 1 mg/L. The limits of detection (LOD) and quantification (LOQ) are reported in Balvis et al. [23].

### 2.6. Volatile Composition of the Substrate and Fermented Beverages

Volatile organic compounds (VOCs) were determined in unfermented orange juice and in the beverages fermented with MKG or WKG after 24 and 48 h of fermentation. VOC extraction and analysis were performed according to the procedure described by Bazán et al. [25].

Briefly, 1 mL of an internal-standard solution of 4-methyl-2-pentanol (1.058 g/L in ethanol) was added to 5 mL of sample. The volatile compounds were extracted with 2 mL of diethyl ether/hexane (1:1, *v*/*v*) by stirring at 300 rpm for 1 min. After standing for 5 min at 20 ± 1 °C, the organic phase was dried over anhydrous sodium sulfate, and 2 µL of the resulting extract was injected into the chromatograph in splitless mode for 30 s.

Separation, identification, and quantification were performed using an Agilent 7820A gas chromatograph (Agilent Technologies, Santa Clara, CA, USA) coupled to a 5975 series mass-selective detector. The system was equipped with a ZB-Wax capillary column (60 m × 0.25 mm internal diameter × 0.25 µm film thickness) from Phenomenex, Alcobendas, Madrid, Spain. The oven temperature was initially maintained at 45 °C for 2 min and then increased at 2 °C/min to 225 °C. The carrier gas was hydrogen at a constant flow rate of 1.2 mL/min, and the injector temperature was 250 °C. Mass spectra were acquired at 70 eV over the *m*/*z* 35–350 range.

VOC identification was based on comparison of mass spectra with authentic standards and the Wiley spectral library, together with retention indices and retention times. When available, pure reference compounds were used to confirm compound identities. Compounds identified solely from mass-spectral data were considered tentatively identified. Quantification was performed using the internal-standard method based on the relative peak area of each compound to that of 4-methyl-2-pentanol. Aliquot C from each of the two independent jars per treatment and time was extracted and analyzed in triplicate.

The contribution of each identified compound to the aroma profile of the beverages was subsequently evaluated using its odor activity value (OAV), calculated as the ratio of the compound concentration to its odor perception threshold (OPT). Compounds with OAV ≥1 were considered potentially relevant contributors to the overall aroma of the beverages [23,25]. Because no sensory panel was used in the present study, OAVs were interpreted as indicators of potential aroma contribution rather than as direct evidence of perceived sensory intensity.

### 2.7. Antibacterial Activity Assay

Total antibacterial activity (TAA) was determined in unfermented orange juice and in fermented substrates collected after 8, 16, 24, 32, and 48 h of fermentation. The assay was performed using a photometric bioassay based on the procedure described by Cabo et al. [28] and the inhibitory dose 50 (ID_50_) approach developed by Murado et al. [29].

For the assay, samples were acidified to pH 3.5 with 5 N HCl and subsequently heated in a boiling-water bath for 3 min to inactivate viable microbial cells. After cooling to room temperature (20 ± 1 °C), the samples were centrifuged at 5000 rpm for 15 min at 4 °C, and the resulting pellets were discarded [28]. The cell-free supernatants were then adjusted to pH 6.0 with 4 N NaOH to minimize the direct contribution of sample acidity to the inhibition of the indicator microorganism.

Antibacterial activity was evaluated against *Carnobacterium piscicola* CECT 4020 (Spanish Type Culture Collection, Paterna, Spain), which was used as the indicator strain. The strain was maintained at −40 °C in Nutrient broth with 15% (*v*/*v*) glycerol and subcultured in MRS broth at 30 °C for 12 h before each assay. The culture was diluted in buffered MRS broth (pH 6.3; 0.05 M sodium biphthalate–NaOH) to an absorbance of 0.2 at 700 nm. Appropriate serial dilutions of each cell-free supernatant were prepared in sterile distilled water. Equal volumes (1 mL) of the diluted samples and indicator culture were mixed in culture tubes and incubated for 6 h at 30 °C with shaking at 200 rpm [28].

Controls consisted of supernatants obtained from unfermented orange juice and subjected to the same sample-treatment procedure, followed by incubation with the indicator strain under identical conditions [28]. After incubation, absorbance was measured at 700 nm. The inhibition index for each supernatant dilution was calculated according to Balvis et al. [23] and plotted against the inverse of the corresponding sample dilution. The inhibitory dose 50 (ID_50_), defined as the dilution producing 50% growth inhibition of the indicator microorganism relative to the corresponding control, was estimated by fitting an appropriate dose–response model to the experimental data [29]. Total antibacterial activity was expressed as antibacterial activity units per milliliter (AU/mL), with the AU defined as the ID_50_ obtained from the corresponding dose–response model.

### 2.8. Statistical Analyses

The mean values of the fermentation variables, including pH; microbial counts of LAB, AAB, yeasts, *Pseudomonas* spp., and Enterobacteriaceae (expressed as log CFU/mL); concentrations of proteins, sugars, organic acids, and alcohols; and total antibacterial activity in unfermented and fermented orange juice samples were statistically compared using one-way analysis of variance (ANOVA) at a significance level of 5%. Tukey’s honestly significant difference (HSD) post hoc test was applied when the variances were homogeneous, whereas the Games–Howell post hoc test was used when the variances were heterogeneous. Homogeneity of variances was assessed using Levene’s test. All analyses were performed using IBM^®^ SPSS^®^ Statistics for Windows, version 25.0 (IBM Corp., Armonk, NY, USA).

The concentrations of vitamins, minerals, and volatile compounds in unfermented orange juice (UOJ) and in beverages fermented with milk kefir grains (MKG) or water kefir grains (WKG) for 24 and 48 h were analyzed using the General Linear Model (GLM) procedure in IBM^®^ SPSS^®^ Statistics for Windows, version 25.0 (IBM Corp., Armonk, NY, USA), with a significance level of 5% (*p* < 0.05). For the fermented beverages, a two-way factorial model was used to evaluate the effects of kefir grain type (KG-TYPE), fermentation time (F-TIME), and their interaction (KG-TYPE × F-TIME) on the concentration of each compound. Exact *p* values, F statistics, effect-size estimates (partial eta squared, ηp^2^), R^2^, and adjusted R^2^ values are reported to facilitate the interpretation of statistical significance, effect magnitude, and model explanatory power. The assumption of homogeneity of variances was assessed using Levene’s test. To perform multiple comparisons among the observed means for the levels of KG-TYPE and F-TIME for each compound, a one-way analysis of variance (ANOVA) was performed when the homogeneity-of-variance assumption was met, followed by Tukey’s honestly significant difference (HSD) post hoc test. When this assumption was violated, Welch’s ANOVA was used instead, followed by the Games–Howell post hoc test. Differences were considered statistically significant at *p* < 0.05.

Because each grain type was evaluated in a single fermentation run with two in-dependent jars per sampling time, the variability reported reflects jar-to-jar and analytical variability within one run; batch-to-batch reproducibility (different grain lots and juice batches) should be confirmed in future studies.

## 3. Results and Discussion

### 3.1. Fermentation Dynamics of Orange Juice with Milk and Water Kefir Grains

**Figure 2:**
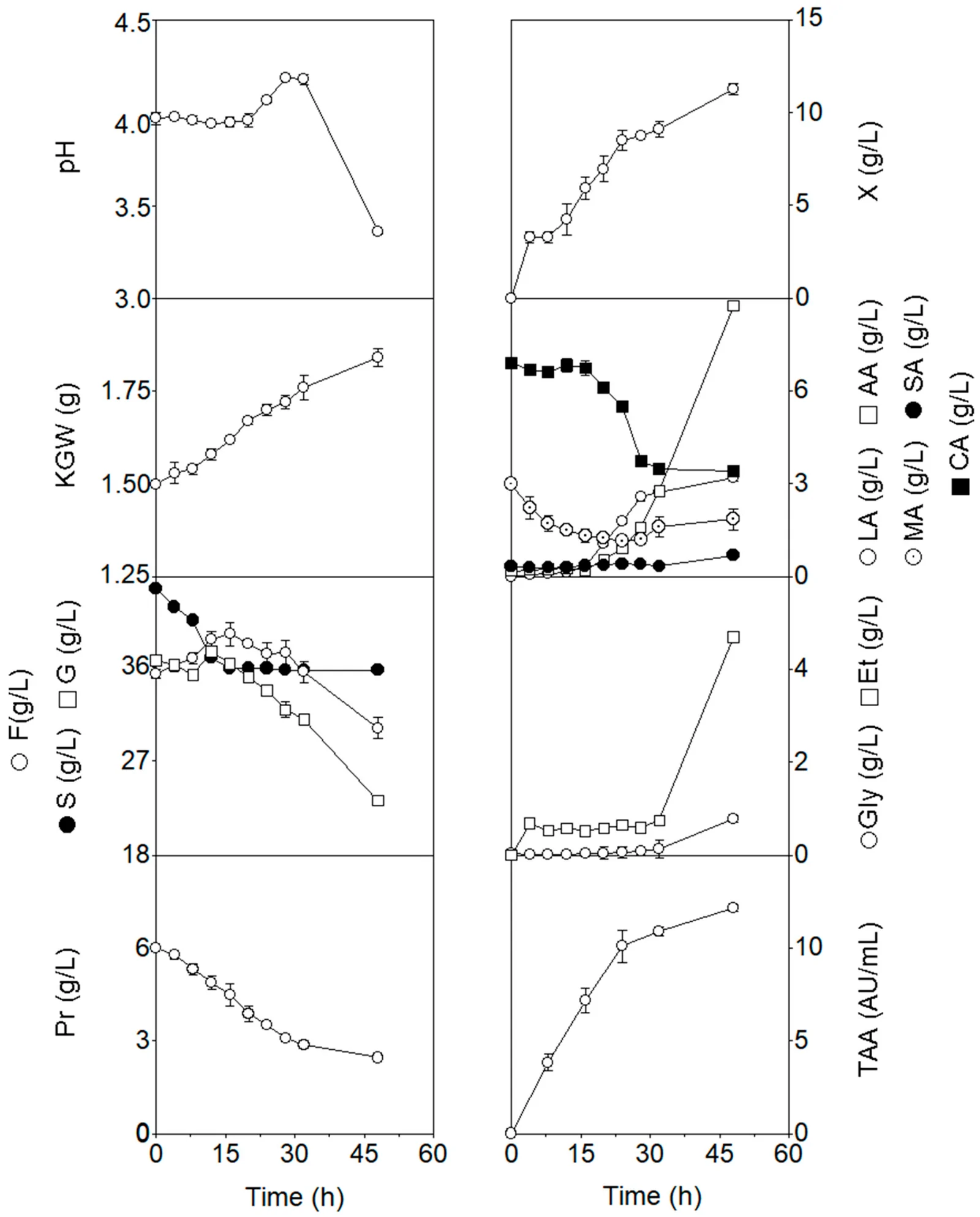
Time course of pH, free biomass in the fermented substrate (X), lactic acid (LA), acetic acid (AA), malic acid (MA), succinic acid (SA), citric acid (CA), kefir grain weight (KGW), fructose (F), sucrose (S), glucose (G), ethanol (Et), glycerol (Gly), proteins (Pr), and total antibacterial activity (TAA) during 48 h of fermentation of orange juice wth milk kefir grains.

**Figure 3:**
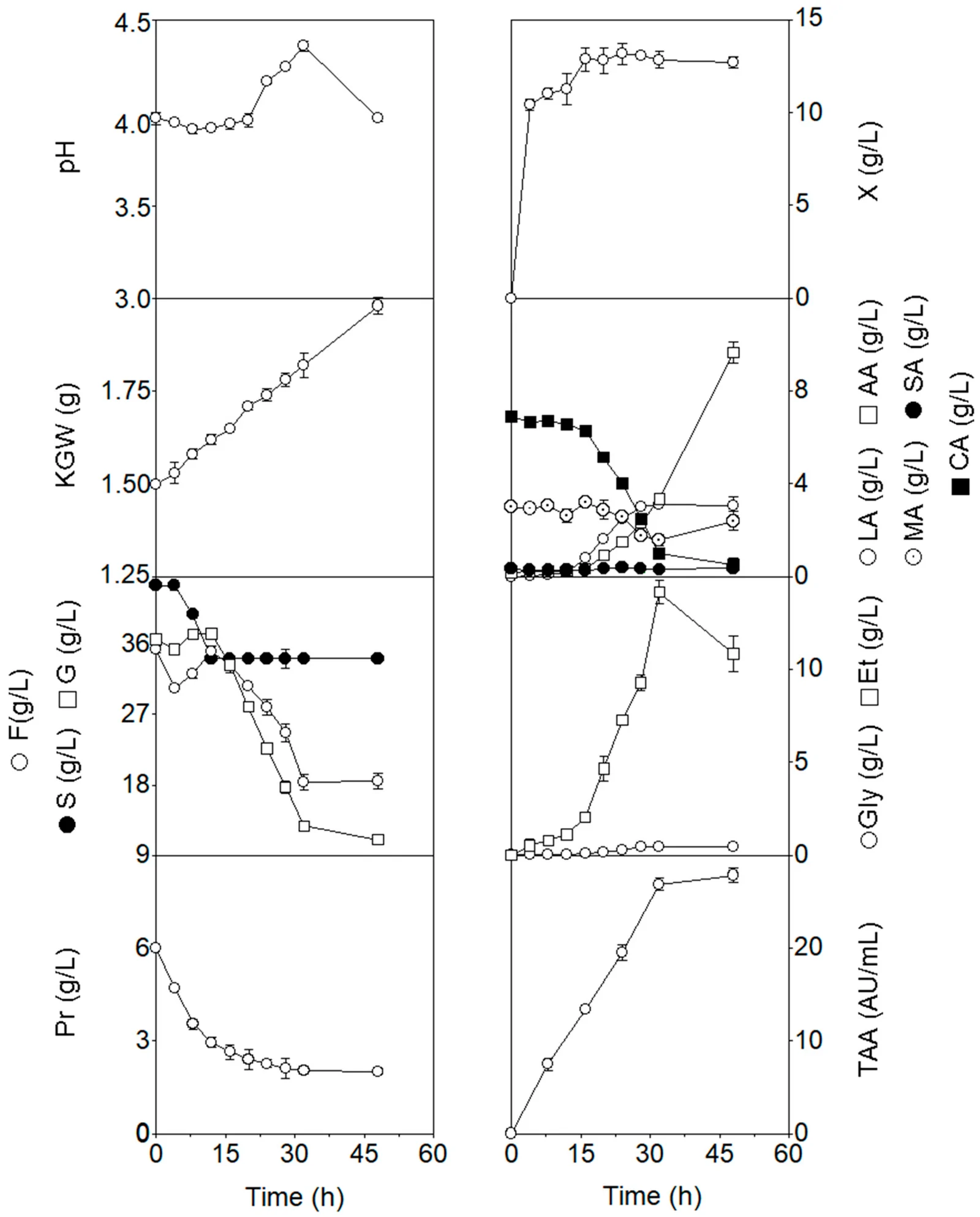
Time course of pH of pH, free biomass in the fermented substrate (X), lactic acid (LA), acetic acid (AA), malic acid (MA), succinic acid (SA), citric acid (CA), kefir grain weight (KGW), fructose (F), sucrose (S), glucose (G), ethanol (Et), glycerol (Gly), proteins (Pr), and total antibacterial activity (TAA) during 48 h of fermentation of orange juice with water kefir grains.

Fermentation of orange juice with milk kefir grains (MKG; Figure 2) and water kefir grains (WKG; Figure 3) resulted in marked biochemical and microbiological changes over the 48 h fermentation period. Although both fermentation systems followed similar overall trends, differences were observed in the magnitude and timing of these changes.

In both systems, pH changed only slightly during the first 32 h (MKG, 3.95–4.19; WKG, 3.91–4.36) and decreased significantly (*p* < 0.05) only at the end of fermentation (MKG, 3.36; WKG, 3.97) (Figure 2 and Figure 3).

Similar slight pH reductions have been reported during the fermentation of kiwi fruit juice with MKG under different agitation rates and inoculum concentrations, with final pH values ranging from 2.98 to 3.17 after fermentation [30]. In contrast, greater pH decreases have been observed in date syrup supplemented with whey permeate and fermented with MKG, where initial pH values decreased from 6.12 to 4.00–4.60 after 48 h [31].

The absence of a decrease in final pH, or even an increase in pH after fermentation, has also been reported for acidic fruit-based substrates fermented with water kefir microorganisms. For example, fermentations of onion, strawberry, and tomato juices, as well as apple, grape, kiwifruit, pomegranate, and quince juices, showed unchanged or increased pH values after 48 h of fermentation with commercial water kefir starters [8,9]. Similarly, no significant pH variation was observed during the fermentation of a honey-based kefir beverage with WKG, suggesting limited acidification under these conditions [32]. However, when substrates with higher initial pH values were fermented with WKG, marked acidification was observed, including carrot, fennel, melon, and prickly pear substrates, which reached final pH values between 4.10 and 4.40 after 48 h [8,9]. Comparable decreases in pH have also been reported during WKG fermentation of sugar solutions, pumpkin purées, palm sap, and fresh lentils [19,33,34,35].

The pH of the substrate depends on the balance among several organic acids and their salts, which confer buffering capacity [36], rather than on lactic acid alone. The stronger acids originally present in the juice (citric acid, pKa1 = 3.13; malic acid, pKa1 = 3.40) were partly consumed, whereas the acids produced by the grains microbiota are weaker (lactic acid, pKa = 3.86; acetic acid, pKa = 4.76) [18]; their replacement therefore limited, or temporarily reversed, acidification between 16 and 32 h, despite the accumulation of lactic and acetic acids (Figure 2 and Figure 3). This effect was more pronounced with WKG, in which citric acid decreased to 0.99 ± 0.03 g/L at 32 h, compared with 3.46 ± 0.13 g/L with MKG, consistent with the higher pH maximum of the WKG fermentation (4.36 ± 0.01 vs. 4.19 ± 0.03). The final pH decrease between 32 and 48 h coincided in both systems with a sharp increase in acetic acid (MKG, from 2.75 ± 0.06 to 8.74 ± 0.07 g/L; WKG, from 3.33 ± 0.03 to 9.64 ± 0.44 g/L) after ethanol had accumulated, suggesting oxidation of yeast-derived ethanol by AAB [18]. The earlier and greater ethanol formation with WKG is consistent with its higher acetic acid concentration at 24 h (1.48 ± 0.04 vs. 0.90 ± 0.01 g/L).

In both fermentations (Figure 2 and Figure 3), lactic acid (LA) and acetic acid (AA) were the predominant organic acids formed, reflecting the metabolic activities of lactic acid bacteria (LAB) [37,38] and acetic acid bacteria (AAB) [39,40], respectively.

The LA and AA concentrations produced during MKG fermentation after 24 h (1.80 ± 0.08 and 0.90 ± 0.01 g/L, respectively) and 48 h (3.18 ± 0.10 and 8.74 ± 0.07 g/L, respectively) fell within the ranges reported for kefir-like beverages produced from non-dairy substrates using MKG. Similarly, during the fermentation of red table grape juice with MKG, maximum LA and AA concentrations of 2.32 g/L and 3.16 g/L, respectively, were obtained at an initial pH (pHi) of 3.99, whereas values of 2.15 g/L and 3.36 g/L, respectively, were reported at a pHi of 5.99 [25]. In contrast, fermentation of palm sap with MKG for 48 h produced a kefir-like beverage containing 9.31 g LA/L and 1.69 g AA/L [34].

During the fermentation of orange juice with WKG (Figure 3), LA concentrations were 2.49 ± 0.08 and 3.02 ± 0.40 g/L after 24 and 48 h, respectively. These concentrations were higher (*p* < 0.001) and slightly lower (*p* = 0.006), respectively, than the corresponding LA concentrations obtained with MKG (Figure 2 and Figure 3).

In WKG fermentation, AA concentrations reached 1.48 ± 0.04 and 9.64 ± 0.44 g/L after 24 and 48 h, respectively. These concentrations were significantly higher than those obtained with MKG at both 24 h (*p* < 0.001) and 48 h (*p* = 0.001), suggesting a greater contribution of AAB to AA production (Figure 2 and Figure 3).

These LA and AA concentrations obtained during WKG fermentation were within or exceeded the ranges reported for other kefir-like beverages. For comparison, Kazakos et al. [24] reported a LA concentration of 1.90 g/L after fermentation of orange juice with WKG, while Dikmetas et al. [41] reported LA concentrations of 3.14 and 4.29 g/L after 24 and 48 h of orange juice fermentation with WKG, respectively, whereas the corresponding AA concentrations were only 0.25 and 0.18 g/L. In other substrates fermented with water kefir cultures, LA concentrations after 48 h ranged from 0.02 to 4.81 g/L and AA concentrations from 0.03 to 1.90 g/L in vegetable juices (carrot, fennel, melon, onion, strawberry, and tomato) [8] and fruit juices (apple, grape, kiwifruit, pomegranate, prickly pear, and quince) [9]. Similarly, fermentation of palm sap with WKG for 48 h produced a kefir-like beverage containing 5.18 g/L of LA and 1.32 g/L of AA [34].

The acetic acid concentrations reached after 48 h are considerably higher than those reported for other fruit-based kefir-like beverages (0.03–3.36 g/L) [8,9,25,34,41] and could confer marked sourness and pungency, potentially reducing consumer acceptance. From this perspective, shorter fermentation times (e.g., 24 h) may provide a more suitable composition. As no sensory evaluation was performed, the sensory implications of these results remain to be established.

Regarding organic acid metabolism, malic acid (MA) and citric acid (CA) generally decreased over time in both systems, suggesting their utilization by the kefir grain microbiota. The consumption of malic acid is typically associated with malolactic or malo-ethanolic fermentation carried out by yeasts [42,43,44,45]. In addition, citric acid can be assimilated by certain lactic acid bacteria [36] that possess genes encoding citrate permease and citrate lyase [46]. These enzymes mediate citrate uptake and its subsequent conversion to oxaloacetate and ultimately pyruvate [47]. However, some yeasts present in kefir grains (e.g., certain *Saccharomyces* strains) can also produce malic acid during alcoholic fermentation [48], which may explain the increase observed at the end of the MKG (Figure 2) and WKG (Figure 3) fermentations.

Dikmetas et al. [41] likewise reported a decrease in malic acid concentration during the fermentation of orange juice with WKG, from 1.37 g/L in the unfermented substrate to 1.26 g/L after 24 h and 0.97 g/L after 48 h of incubation. The authors also observed an increase in citric acid concentration after 24 h (26.85 g/L), followed by a decrease after 48 h (19.72 g/L) relative to the unfermented substrate (19.35 g/L). Likewise, Bazán et al. [30] reported a reduction in citric acid concentration during the fermentation of kiwi juice with MKG over three consecutive 24-h batch subcultures, with the extent of the reduction depending on inoculum concentration and agitation speed.

At the same time, succinic acid (SA) increased slightly in both MKG and WKG fermentations, consistent with yeast metabolism and potentially contributing to flavor complexity [49].

Sugar metabolism differed markedly between the two fermentations. Sucrose (S) was rapidly hydrolyzed into glucose (G) and fructose (F), followed by the progressive consumption of both monosaccharides. MKG showed a relatively balanced utilization of glucose and fructose, whereas WKG preferentially consumed glucose, resulting in transient fructose accumulation. Sugar consumption was accompanied by ethanol (Et) and glycerol (Gly) formation, with ethanol production being more pronounced during WKG fermentation, consistent with greater yeast activity [6,8,9,19,50]. These differences suggest that the yeast community associated with WKG is better adapted to acidic substrates such as orange juice (pH 3.97) than that associated with MKG, which naturally develops in milk (pH approximately 6.7) [18].

The ethanol concentrations measured in the MKG- and WKG-fermented beverages after 24 h (0.64 ± 0.12 and 7.25 ± 0.04 g/L, respectively) and 48 h (4.69 ± 0.08 and 10.84 ± 0.06 g/L, respectively) corresponded to 0.08%, 0.92%, 0.59%, and 1.37% (*v*/*v*), respectively.

These values are technologically and commercially relevant. In the European Union, beverages containing more than 1.2% (*v*/*v*) alcohol must declare their actual alcoholic strength (Regulation (EU) No 1169/2011, Article 9(1)(k) and Annex XII) [51], a threshold exceeded by the WKG beverage after 48 h. Moreover, both WKG beverages exceeded the 0.5% (*v*/*v*) limit used in several jurisdictions (e.g., the Czech Republic, Denmark, and Ireland) [52] to define non-alcoholic beverages. Ethanol at these levels could limit the suitability of the beverages for children, pregnant women, and consumers who avoid alcohol for health, religious, or personal reasons, and may also contribute to their sensory profile. Therefore, grain type and fermentation time should be selected according to the desired ethanol content. Product development strategies should include shortening the fermentation period, cooling the beverage once the target acidity is reached, or selecting low-ethanol-producing microbial consortia.

These concentrations were comparable to, or lower than, those reported for other non-dairy kefir-like beverages. For example, fermentation of orange juice with MKG for 20 h resulted in an ethanol concentration of 3.94 g/L [24]. Similarly, fermentation of red table grape juice with MKG at initial pH values of 3.99 and 5.99 yielded ethanol concentrations of 21.25 and 28.23 g Et/L, respectively [25], whereas fermentation of palm sap with MKG resulted in 16.61 g Et/L [34].

The substantially higher ethanol production observed during WKG fermentation compared with MKG fermentation under the conditions used in the present study highlights the importance of kefir grain type in determining the alcoholic profile of the resulting beverages.

Regarding WKG, Dikmetas et al. [41] reported ethanol concentrations of 0.65 and 5.60 g/L after 24 and 48 h of orange juice fermentation, respectively. Similarly, ethanol concentrations ranging from 0.71 to 39.13 g/L were reported after 48 h of fermentation of vegetable juices (carrot, fennel, melon, onion, strawberry, and tomato) [8] and fruit juices (apple, grape, kiwifruit, pomegranate, prickly pear, and quince) [9] using a commercial water kefir culture. Furthermore, fermentation of palm sap with WKG for 48 h produced 17.30 g/L [34].

The glycerol (Gly) concentrations measured after 24 and 48 h in the MKG-fermented beverages (0.06 ± 0.02 and 0.78 ± 0.02 g Gly/L, respectively) and WKG-fermented beverages (0.27 ± 0.06 and 0.45 ± 0.03 g Gly/L, respectively) were substantially lower than those reported for red table grape kefir-like beverages fermented with MKG (approximately 9.00 g/L at an initial pH of 3.99 and 10.00 g/L at an initial pH of 5.99) [25]. However, glycerol concentrations have not been reported for kefir-like beverages produced from vegetable juices [8], fruit juices [9], or palm sap [34].

Additionally, variations in the concentrations of organic acids and alcohols during substrate fermentation by MKG and WKG result from their production and consumption by the complex microbiota of the grains [30,45].

Sugar consumption reduced the residual sugar content of the fermented beverages. However, part of the sugars was converted into ethanol, which also contributes energy (approximately 7 kcal/g, compared with approximately 4 kcal/g for sugars); therefore, no conclusion regarding energy content or health benefits can be drawn without a complete compositional and energy assessment.

Free biomass in the fermented substrate increased throughout fermentation in both systems, although the growth patterns differed, indicating microbial proliferation and/or detachment of microorganisms from the kefir grains into the fermentation medium (Figure 2 and Figure 3). During MKG fermentation, free biomass concentration followed a sigmoidal pattern, reaching 11.28 g/L at the end of incubation. In contrast, during WKG fermentation, free biomass increased rapidly to 10.44 g/L within the first 4 h and then continued to increase more gradually, reaching 12.67 g/L after 48 h. This release of microorganisms from both types of kefir grains increased the concentration of viable microorganisms, especially LAB and yeasts, in the fermented beverages [8,9,23].

Similar levels of free biomass have been reported in other kefir-like beverages. For example, Plessas et al. [26] reported free biomass concentrations ranging from 2.9 to 10.6 g/L in a kefir-like beverage produced by fermenting molasses-supplemented orange pulp suspensions with a commercial kefir culture composed of yeasts, lactobacilli, and lactococci for 21.8–25.5 h.

The greater increase in kefir grain weight (KGW) observed for WKG (1.50–1.98 g) suggests that the water kefir consortium was better adapted to the orange juice matrix or produced larger amounts of extracellular polysaccharides under these conditions [53,54,55]. In contrast, MKG showed more limited growth (from 1.50 to 1.84 g), possibly reflecting lower substrate compatibility.

Protein (Pr) concentration decreased during both fermentations, likely as a result of microbial proteolysis and acid-induced protein denaturation and precipitation associated with LAB activity [56]. The decrease was similar in both systems, with protein concentrations declining from 6.00 ± 0.11 g/L to 2.46 ± 0.02 g/L in the MKG fermentation and to 2.02 ± 0.10 g/L in the WKG fermentation by the end of the incubation period. This reduction in protein concentration during fermentation is consistent with previous results obtained in milk fermented with the same kefir grains [23], but contrasts with the increase in protein concentration reported by Alves et al. [57] after 24 h of fermentation of a water-soluble coconut extract with water kefir grains.

Finally, total antibacterial activity (TAA) against the indicator strain *C. piscicola* CECT 4020 increased during fermentation in both systems, with higher values for WKG. Because the supernatants were heated and adjusted to pH 6.0 before the assay, the activity detected cannot be ascribed to undissociated organic acids at the pH of the beverages or to ethanol, and is more likely associated with other heat-stable metabolites produced by the grain microbiota [30]. These results demonstrate in vitro antibacterial activity against a single indicator strain; demonstrating improved product safety would require challenge tests with relevant foodborne pathogens in the beverages [58].

Overall, both milk and water kefir grains efficiently fermented orange juice but produced beverages with distinct metabolic profiles. MKG fermentation was characterized by slower initial lactic acid production (24 h) but a slightly higher final concentration (48 h), whereas WKG fermentation favored earlier lactic acid formation and higher antibacterial activity, acetic acid and ethanol production. These differences most likely reflect the distinct microbial communities and metabolic interactions associated with each type of kefir grain.

### 3.2. Population Dynamics of Viable LAB, AAB, and Yeasts During MKG and WKG Fermentations

**Figure 4:**
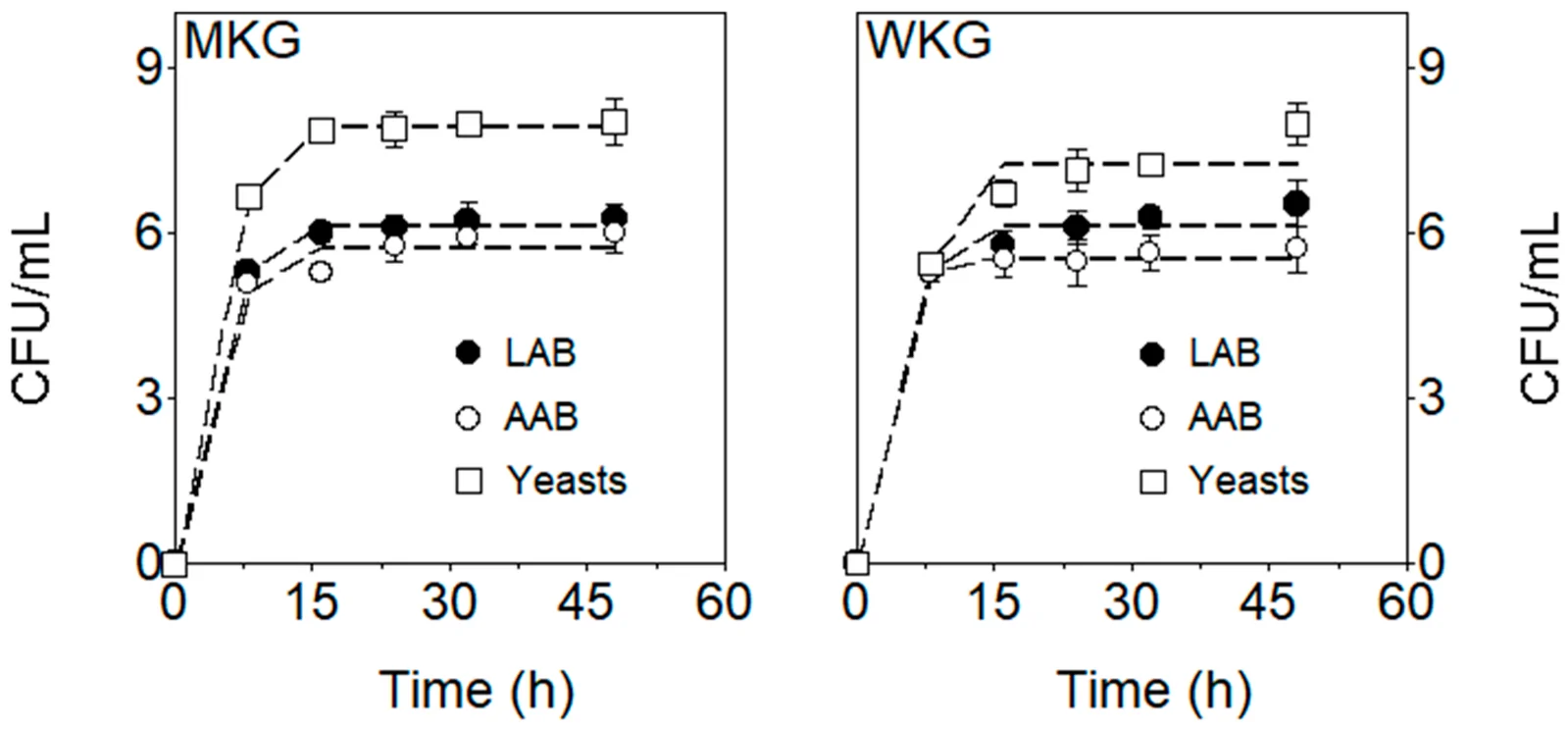
Growth of lactic acid bacteria (LAB), acetic acid bacteria (AAB), and yeasts (expressed as colony-forming units (CFU) per mL) in orange juice fermented with milk kefir grains (MKG) and water kefir grains (WKG).

Figure 4 shows the changes in viable counts of lactic acid bacteria (LAB), acetic acid bacteria (AAB), and yeasts during orange juice fermentation with milk kefir grains (MKG) and water kefir grains (WKG). In both fermentations, all microbial groups increased rap-idly during the early stages of incubation before reaching a stationary phase, indicating adaptation of the kefir-associated microbiota to the orange juice matrix.

During MKG fermentation, LAB populations increased, reaching 6.01 log CFU/mL after 16 h and remaining relatively stable thereafter, with a final population of 6.27 log CFU/mL at 48 h. AAB exhibited a similar growth pattern but remained slightly less abundant than LAB throughout fermentation, stabilizing between 5.77 and 6.02 log CFU/mL from 24 to 48 h. Yeasts were the predominant microbial group, increasing rapidly to 7.88 log CFU/mL after 16 h and maintaining high population levels throughout the remainder of the fermentation, reaching 8.02 log CFU/mL at 48 h.

A similar growth pattern was observed during WKG fermentation, although some differences in population dynamics were evident. LAB increased rapidly during the first 16 h, reaching 6.53 log CFU/mL, slightly higher than the final LAB population observed during MKG fermentation. AAB populations remained lower, stabilizing between 5.47 and 5.72 log CFU/mL, indicating growth of AAB in the orange juice matrix. As observed during MKG fermentation, yeasts were the dominant microbial group, reaching approximately 7.98 log CFU/mL after 48 h. Overall, yeast populations were very similar in the two fermentations (Figure 4).

These results show that both MKG- and WKG-fermented beverages contained viable LAB populations of approximately 10^6^–10^7^ CFU/mL after 24 and 48 h of fermentation, whereas yeast populations reached approximately 10^8^ CFU/mL. The presence of these viable microbial groups is characteristic of kefir fermentation and indicates that LAB, AAB, and yeasts remained viable throughout the fermentation process. However, because the microorganisms were quantified only at the microbial-group level, without species- or strain-level identification or assessment of probiotic properties, the present results do not allow the fermented beverages to be classified as probiotic [8,9,59] or their potential health effects to be established.

Comparable microbial populations have been reported for kefir fermentation of orange juice. Kazakos et al. [24] reported LAB populations of 8.38 log CFU/mL after 20 h of fermentation with MKG. During orange juice fermentation with WKG, Dikmetas et al. [41] reported lactobacilli populations of approximately 4.41 log CFU/mL after both 24 and 48 h, whereas lactococci reached 4.41 and 5.33 log CFU/mL after 24 and 48 h, respectively. Yeast populations reached 5.56 and 7.56 log CFU/mL after 24 and 48 h, respectively [41]. However, neither study quantified AAB populations [24,41], and Kazakos et al. [24] did not enumerate yeasts, limiting direct comparisons among the different microbial groups.

Overall, both MKG and WKG supported rapid proliferation of the quantified microbial groups in orange juice during the first 16 h of fermentation, after which the populations entered a relatively stable phase (Figure 4). The predominance of yeasts over bacteria suggests that yeasts were well adapted to the acidic conditions of orange juice (pH 3.36–4.36) [60]. The growth of LAB, AAB, and yeasts may have contributed to acidification and flavour development; however, the present data do not allow specific metabolic interactions among these microbial groups to be established. The small differences observed in microbial populations between the MKG- and WKG-fermented beverages may be related to differences in the initial microbiota associated with each type of kefir grain [6,7,18,23]. Nevertheless, because only broad microbial groups were quantified, differences in the species or strains responsible for these population patterns cannot be determined from the present data.

*Pseudomonas* spp. and Enterobacteriaceae counts remained below 30 CFU/mL (1.48 log CFU/mL) in the unfermented orange juice and all fermented samples. These low counts may indicate the microbiological quality of both the unfermented and fermented beverages; however, they cannot, by themselves, replace a comprehensive assessment of food safety.

Because the orange juice was not heat-treated, the enumerated populations may have included a minor fraction of indigenous microorganisms from the juice. The initial counts of LAB, AAB, and yeasts in the unfermented juice (all below 1.48 log CFU/mL) were lower than those reached after 8 h of fermentation, suggesting that the grain-derived microbiota most likely predominated. However, distinguishing between these two microbial sources would require molecular identification, which was beyond the scope of this study.

### 3.3. Changes in Vitamin Composition During Fermentation with Milk and Water Kefir Grains

**Figure 5:**
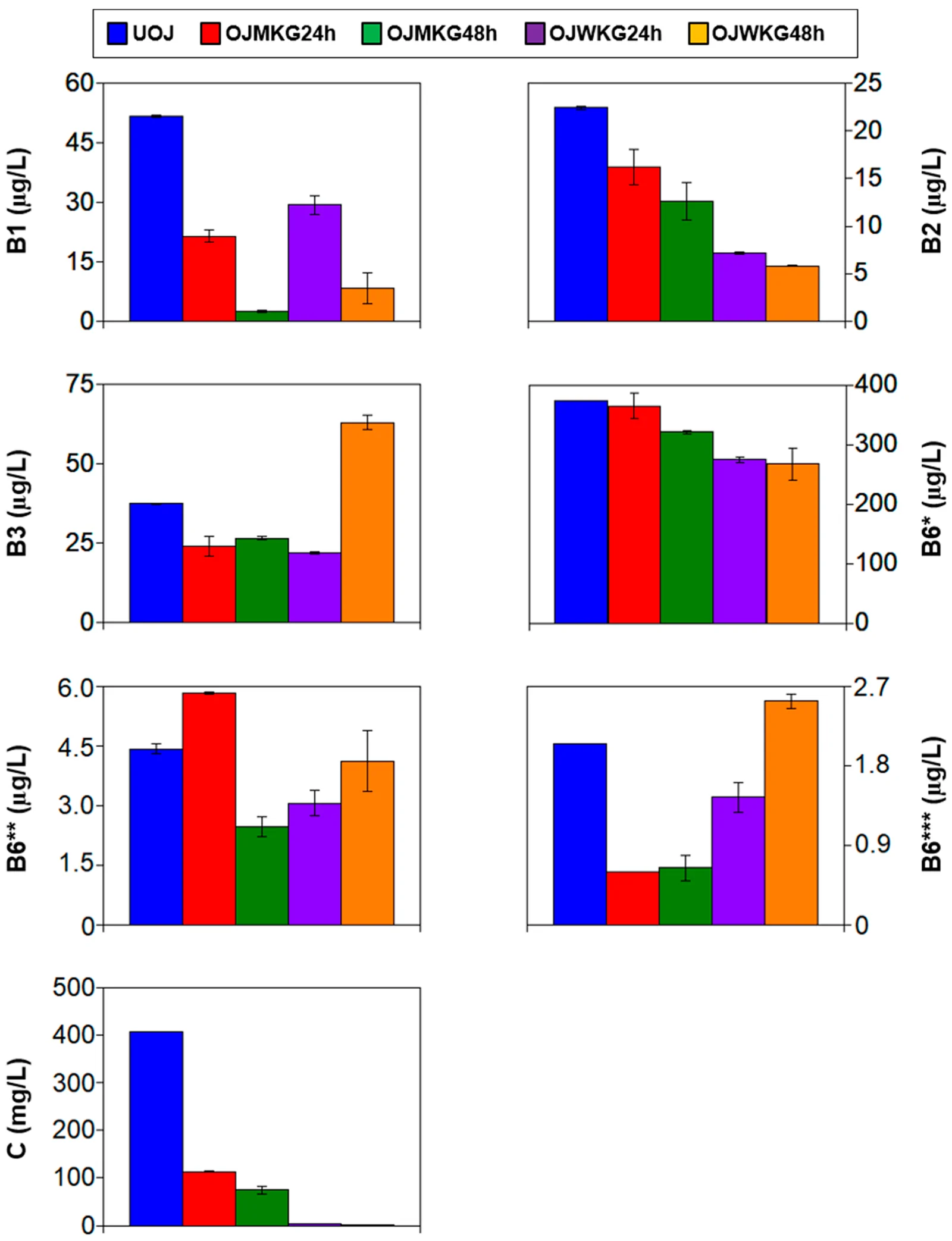
Vitamin concentrations (mean ± standard deviation) in unfermented orange juice (UOJ) and orange juice fermented with milk kefir grains (OJMKG24h and OJMKG48h) or water kefir grains (OJWKG24h and OJWKG48h) for 24 and 48 h. Values represent two independent fermentation jars (*n* = 2), each analyzed in triplicate. B6*: pyridoxal; B6**: pyridoxine; B6***: pyridoxamine.

Figure 5 shows the vitamin concentrations in unfermented orange juice (UOJ) and in orange juice fermented with milk kefir grains (MKG) or water kefir grains (WKG) for 24 and 48 h. The effects of kefir grain type (KG-TYPE), fermentation time (F-TIME), and their interaction (KG-TYPE × F-TIME) on individual vitamin concentrations were evaluated using a general linear model (GLM), with the corresponding results presented in Table S1.

Information on changes in the concentrations of thiamine (vitamin B1), riboflavin (vitamin B2), niacin (vitamin B3), and the three vitamin B6 vitamers (pyridoxal, pyridoxine, and pyridoxamine) during storage of orange juice at room temperature in the absence of light is limited. Therefore, changes observed during fermentation should be interpreted considering both the potential effects of these storage conditions on vitamin stability and the metabolic activity of the microorganisms present in milk and water kefir grains. In particular, kefir microorganisms can utilize [61,62,63,64,65,66], synthesize [20,62,67,68], or transform [66] some of these vitamins, potentially contributing to the changes in their concentrations observed throughout fermentation.

Thiamine decreased markedly during both fermentations (Figure 5). During MKG fermentation, its concentration declined from 51.55 ± 0.29 µg/L in UOJ to 21.44 ± 1.53 µg/L after 24 h and to 2.48 ± 0.22 µg/L after 48 h. A similar trend was observed during WKG fermentation, although the concentrations remaining after 24 and 48 h were slightly higher (29.32 ± 2.46 and 8.34 ± 3.76 µg/L, respectively). This decrease may be related to the utilization of thiamine by yeasts and LAB, for which it serves as an essential cofactor in carbohydrate metabolism and microbial growth [61,62].

The GLM for vitamin B1 (Table S1) showed an excellent fit (R^2^ = 0.988, adjusted R^2^ = 0.986, F = 494.27, *p* < 0.001), explaining 98.8% of the observed variability. Both KG-TYPE (F = 62.53, *p* < 0.001, ηp^2^ = 0.714) and F-TIME (F = 528.28, *p* < 0.001, ηp^2^ = 0.955) significantly affected vitamin B1 concentration, whereas their interaction was not significant (F = 1.36, *p* = 0.255). Thus, fermentation time had a similar effect on vitamin B1 concentration in both kefir grain types.

Riboflavin concentration decreased from 22.40 ± 0.19 µg/L in UOJ to 16.18 ± 1.88 and 12.63 ± 1.98 µg/L after 24 and 48 h of MKG fermentation, respectively. WKG fermentation resulted in lower concentrations, reaching 7.16 ± 1.47 and 5.81 ± 1.11 µg/L at the same time points (Figure 5), which may reflect greater riboflavin utilization by the water kefir microbiota [64,65,66], although non-microbial degradation during incubation cannot be excluded. The GLM (Table S1) showed a strong fit (R^2^ = 0.953, adjusted R^2^ = 0.946, F = 127.03, *p* < 0.001). KG-TYPE (F = 172.90, *p* < 0.001, ηp^2^ = 0.874) had a substantially greater effect on riboflavin concentration than F-TIME (F = 16.56, *p* < 0.001, ηp^2^ = 0.399), whereas their interaction was not significant (F = 3.33, *p* = 0.080). Therefore, differences between kefir grain types were more influential than fermentation time in determining riboflavin concentration.

Niacin exhibited a distinct pattern. During MKG fermentation, its concentration decreased from 37.42 ± 0.15 to 24.00 ± 3.25 µg/L after 24 h and then increased slightly to 26.45 ± 0.46 µg/L after 48 h, remaining below the initial level. In contrast, WKG fermentation resulted in an initial decrease to 21.87 ± 0.40 µg/L after 24 h, followed by a marked increase to 62.89 ± 2.23 µg/L after 48 h, exceeding the concentration in UOJ (Figure 5). The initial decrease may reflect microbial utilization for cofactor biosynthesis, particularly by yeasts [67], whereas the subsequent increase is consistent with niacin production by certain LAB [20,62,67,68]. These contrasting patterns suggest that the microbial composition of the kefir grains plays an important role in vitamin B3 metabolism during fermentation. The GLM for vitamin B3 (Table S1) showed an excellent fit (R^2^ = 0.989, adjusted R^2^ = 0.987, F = 540.99, *p* < 0.001). KG-TYPE (F = 554.14, *p* < 0.001, ηp^2^ = 0.957), F-TIME (F = 889.70, *p* < 0.001, ηp^2^ = 0.973), and their interaction (F = 700.40, *p* < 0.001, ηp^2^ = 0.966) were all significant, with large effect sizes. The significant interaction indicates that changes in vitamin B3 concentration over time depended strongly on kefir grain type, consistent with the contrasting temporal patterns observed for MKG and WKG fermentation.

The three vitamin B6 vitamers showed distinct responses to fermentation. Pyridoxal, the predominant vitamer, remained relatively stable after 24 h of MKG fermentation (364.95 ± 21.14 µg/L) but decreased to 321.10 ± 2.40 µg/L after 48 h compared with 374.00 ± 0.20 µg/L in UOJ. During WKG fermentation, the decrease was more pronounced and occurred mainly during the first 24 h, reaching 274.95 ± 4.60 µg/L and subsequently 267.40 ± 27.29 µg/L after 48 h (Figure 5).

The GLM for B6-pyridoxal (Table S1) showed a strong fit (R^2^ = 0.906, adjusted R^2^ = 0.890, F = 59.91, *p* < 0.001). KG-TYPE (F = 127.09, *p* < 0.001, ηp^2^ = 0.836), F-TIME (F = 16.26, *p* < 0.001, ηp^2^ = 0.394), and their interaction (F = 8.11, *p* < 0.001) significantly affected pyridoxal concentration. The larger effect size for KG-TYPE indicates that grain type was the main determinant of pyridoxal concentration, while the significant interaction shows that its temporal pattern also depended on the fermentation system. The decrease in pyridoxal concentration over time was less pronounced during MKG than WKG fermentation (Figure 5).

Pyridoxine showed a different temporal response. During MKG fermentation, its concentration increased from 4.41 ± 0.12 µg/L in UOJ to 5.82 ± 0.03 µg/L after 24 h, suggesting possible microbial synthesis [67], before declining to 2.46 ± 0.26 µg/L after 48 h (Figure 5). During WKG fermentation, pyridoxine decreased slightly to 3.05 ± 0.32 µg/L after 24 h but subsequently increased to 4.11 ± 0.76 µg/L after 48 h.

The GLM for B6-pyridoxine (Table S1) showed a strong fit (R^2^ = 0.914, adjusted R^2^ = 0.900, F = 66.60, *p* < 0.001). KG-TYPE (F = 12.46, *p* = 0.002, ηp^2^ = 0.333), F-TIME (F = 52.37, *p* < 0.001, ηp^2^ = 0.677), and their interaction (F = 192.09, *p* < 0.001, ηp^2^ = 0.885) were significant. The interaction had the largest effect, indicating that changes in pyridoxine concentration over time were strongly dependent on kefir grain type.

Pyridoxamine exhibited a contrasting pattern. During MKG fermentation, its concentration decreased markedly from 2.04 ± 0.00 µg/L in UOJ to 0.60 ± 0.00 µg/L after 24 h and 0.64 ± 0.14 µg/L after 48 h. During WKG fermentation, the decrease was less pronounced after 24 h (1.44 ± 0.17 µg/L), followed by an increase to 2.53 ± 0.08 µg/L after 48 h. These differences, together with the patterns observed for pyridoxal and pyridoxine, are consistent with microbial interconversion among vitamin B6 vitamers [66].

The GLM for B6-pyridoxamine (Table S1) showed a strong fit (R^2^ = 0.985, adjusted R^2^ = 0.983, F = 409.22, *p* < 0.001), with significant effects of KG-TYPE (F = 1057.51, *p* < 0.001, ηp^2^ = 0.977), F-TIME (F = 179.97, *p* < 0.001, ηp^2^ = 0.878), and their interaction (F = 155.29, *p* < 0.001, ηp^2^ = 0.861). Thus, pyridoxamine changed differently in the two systems, with a smaller increase from 24 to 48 h in MKG-fermented samples.

Comparable decreases in B-group vitamins have been reported in fermented fruit- and plant-based substrates, reflecting strain-specific differences in the balance between microbial vitamin biosynthesis and utilization during fermentation [69].

Among all the vitamins analysed, vitamin C showed the greatest decrease in concentration. Its concentration decreased from 405.35 ± 0.01 mg/L in UOJ to 113.19 ± 0.53 and 74.25 ± 7.57 mg/L after 24 and 48 h of MKG fermentation, respectively. WKG fermentation resulted in an even greater decrease, with concentrations of only 3.25 ± 0.67 and 1.61 ± 0.02 mg/L after 24 and 48 h, respectively. Because a time-matched UOJ control was not included in this study, the observed decrease cannot be unequivocally attributed to a specific mechanism. It may be associated with the inherent instability and oxidative degradation of vitamin C [69,70,71], microbial utilization by LAB [69,72,73,74] and yeasts [75,76], or a combination of these factors. Similar decreases in vitamin C concentration have been reported in other fruit-based fermented beverages [74,77,78,79,80,81].

The GLM for vitamin C (Table S1) showed an excellent fit (R^2^ = 1.000, adjusted R^2^ = 0.999, F = 14,371.58, *p* < 0.001). KG-TYPE had the largest effect (F = 4307.63, *p* < 0.001, ηp^2^ = 0.994), followed by F-TIME (F = 212.95, *p* < 0.001, ηp^2^ = 0.895) and their interaction (F = 180.02, *p* < 0.001, ηp^2^ = 0.878). This interaction reflects the different time-dependent vitamin C profiles of the two grain types; KG-TYPE accounted for the largest share of explained variability.

Overall, the fermented samples showed lower concentrations of vitamins B1, B2, and C than the initial orange juice. However, the increase in vitamin B3 after 48 h of WKG fermentation and the transient accumulation of pyridoxine during MKG fermentation may indicate that microbial vitamin biosynthesis or transformation occurred during fermentation. Among the conditions evaluated, 24 h of MKG fermentation generally resulted in higher vitamin concentrations than the other fermented samples. These findings suggest that kefir grain type and fermentation time influence vitamin composition, potentially through differences in microbial utilization, biosynthesis, and transformation [68,74,82]. However, because time-matched uninoculated controls were not analyzed, the observed changes cannot be attributed solely to kefir microbial activity, as vitamin degradation or transformation during incubation and storage may also have contributed. The distinct responses observed between MKG and WKG may therefore reflect differences in the microbial consortia, although the contribution of incubation-related changes cannot be excluded.

Previous studies of fruit-based kefir beverages have focused primarily on microbiological, physicochemical, and phenolic characteristics, and direct comparisons of vitamin composition remain scarce. To our knowledge, this is the first study to simultaneously quantify vitamins B1, B2, B3, the three vitamin B6 vitamers, and C in a fruit-based kefir beverage. These findings provide new insights into the effects of milk and water kefir grain fermentation on the vitamin composition of orange juice.

### 3.4. Changes in Mineral Composition During Fermentation with Milk and Water Kefir Grains

**Figure 6:**
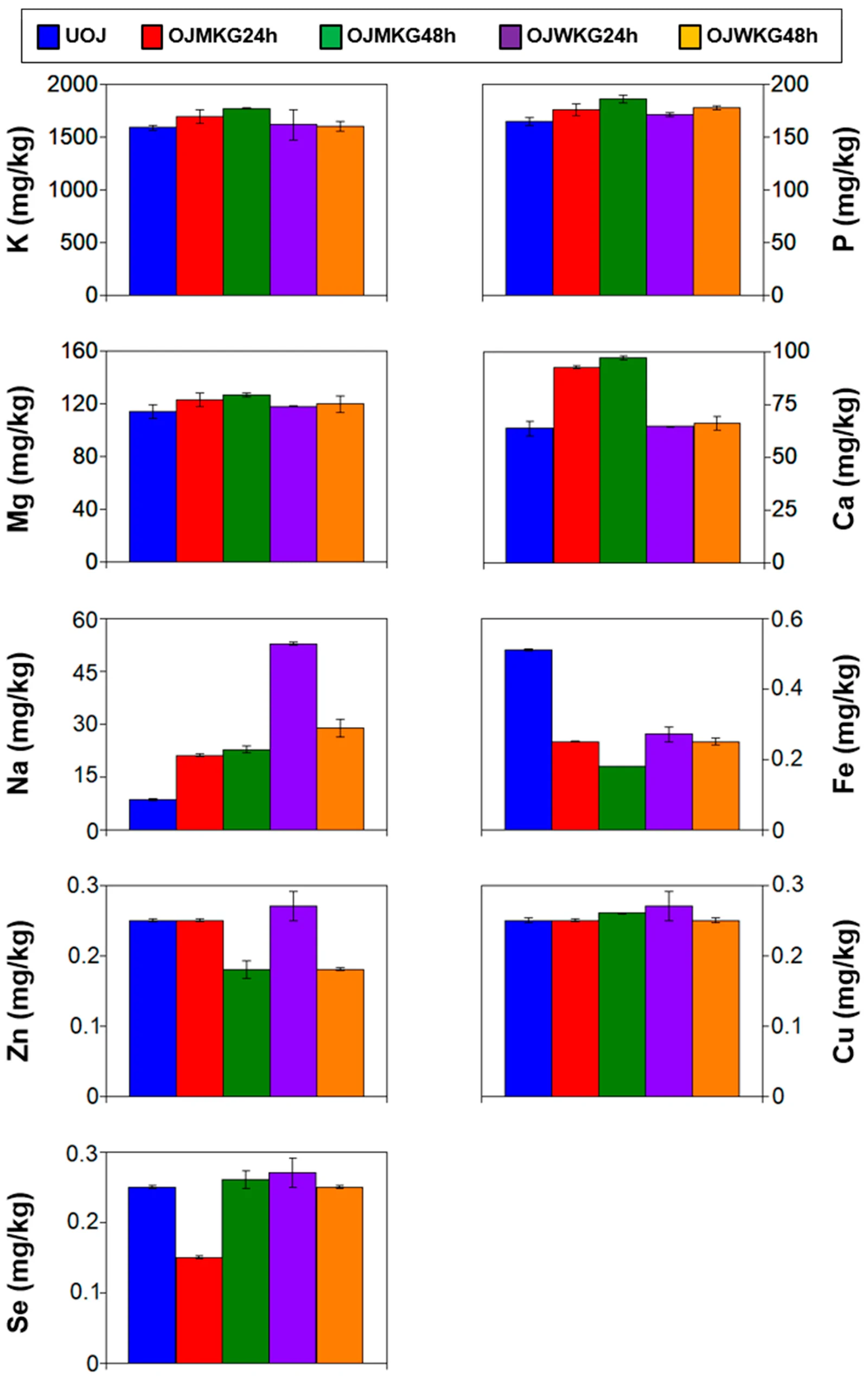
Mineral concentrations (mean ± standard deviation of two independent fermentation jars (n = 2), each analyzed in triplicate) in unfermented orange juice (UOJ) and in orange juice fermented with milk kefir grains (OJMKG24h and OJMKG48h) or water kefir grains (OJWKG24h and OJWKG48h) for 24 and 48 h.

Figure 6 shows the mineral composition of orange juice before and after fermentation with milk kefir grains (MKG) and water kefir grains (WKG). Fermentation significantly affected mineral concentrations, although the magnitude and direction of these changes depended on the mineral, kefir grain type, and fermentation time.

Potassium, the predominant mineral in all samples, increased throughout MKG fermentation, rising from 1590.96 ± 22.45 mg/kg in unfermented orange juice (UOJ) to 1696.55 ± 63.77 and 1772.12 ± 6.64 mg/kg after 24 and 48 h, respectively. In contrast, WKG fermentation produced smaller increases, reaching 1616.04 ± 141.64 and 1600.33 ± 47.61 mg/kg after 24 and 48 h, respectively, suggesting a lower contribution of water kefir grains to potassium release.

The GLM analysis (R^2^ = 0.517; adjusted R^2^ = 0.440; F = 6.69, *p* = 0.001) corroborated these observations and revealed that KG-TYPE had a significant and relatively large effect on K concentration (F = 17.72, *p* < 0.001, ηp^2^ = 0.415), whereas F-TIME (F = 1.00, *p* = 0.327, ηp^2^ = 0.038) and the KG-TYPE × F-TIME interaction (F = 2.32, *p* = 0.140, ηp^2^ = 0.085) were not significant (Table S2). These results indicated that potassium concentration was significantly higher in MKG-fermented samples than in both WKG-fermented and unfermented samples, whereas K concentrations did not differ significantly between the latter two groups. Fermentation time (24 vs. 48 h) did not significantly affect K concentration, nor did its effect differ according to kefir grain type.

Phosphorus followed a similar pattern, increasing during MKG fermentation from 164.82 ± 3.29 mg/kg in the unfermented juice to 176.03 ± 5.63 and 186.18 ± 3.50 mg/kg after 24 and 48 h, respectively. During WKG fermentation, phosphorus concentrations also increased, reaching 171.14 ± 1.68 and 177.85 ± 1.70 mg/kg after the corresponding fermentation times. The GLM analysis (Table S2) indicated that P concentration was significantly affected by KG-TYPE (F = 21.65, *p* < 0.001, ηp^2^ = 0.464) and F-TIME (F = 35.26, *p* < 0.001, ηp^2^ = 0.585). In contrast, the KG-TYPE × F-TIME interaction was not significant (F = 1.46, *p* = 0.238, ηp^2^ = 0.055), indicating that the effect of fermentation time did not significantly differ among KG-TYPE treatments.

Magnesium increased slightly during MKG fermentation, from 113.93 ± 5.21 mg/kg in UOJ to 122.68 ± 5.22 and 126.89 ± 1.47 mg/kg after 24 and 48 h, respectively. In contrast, its concentration remained relatively stable during WKG fermentation (118.07 ± 0.35–119.73 ± 6.39 mg/kg), suggesting a balance between mineral release and microbial assimilation. The GLM analysis (Table S2) revealed a significant effect of KG-TYPE on Mg concentration (F = 10.66, *p* = 0.003, ηp^2^ = 0.299). Post-hoc comparisons showed that Mg concentrations were significantly higher in MKG-fermented samples than in WKG-fermented and unfermented juice, whereas the difference between the latter two groups was not significant. In contrast, neither F-TIME (F = 2.65, *p* = 0.116, ηp^2^ = 0.096) nor the KG-TYPE × F-TIME interaction (F = 0.50, *p* = 0.487, ηp^2^ = 0.020) was statistically significant. Thus, there was no evidence that the effect of fermentation time on Mg concentration depended on KG-TYPE; equivalently, the differences among KG-TYPE groups did not change significantly between 24 and 48 h.

Calcium exhibited the greatest increase during MKG fermentation, rising from 63.66 ± 3.43 mg/kg in UOJ to 92.75 ± 0.90 and 97.15 ± 0.82 mg/kg after 24 and 48 h, respectively, suggesting substantial calcium release from the milk kefir grains. Conversely, calcium concentrations remained essentially unchanged during WKG fermentation (64.60 ± 0.14–66.15 ± 3.19 mg/kg). The statistical analysis (Table S2) indicated that kefir grain type was the dominant determinant of Ca concentration (F = 1119.49, *p* < 0.001, ηp^2^ = 0.978), with MKG fermentation associated with markedly higher Ca levels than both WKG fermentation and unfermented juice. In contrast, no significant difference was observed between UOJ and WKG-fermented samples. Thus, the overall pattern was MKG > WKG ≈ UOJ. Fermentation time also had a significant effect (F = 11.34, *p* = 0.002, ηp^2^ = 0.312), indicating that extending fermentation from 24 to 48 h produced a smaller but statistically significant increase (Figure 6). However, the nonsignificant KG-TYPE × F-TIME interaction (F = 2.61, *p* = 0.119, ηp^2^ = 0.094) indicated that the magnitude of the temporal change did not differ significantly between MKG and WKG.

Sodium exhibited a different pattern. During MKG fermentation, its concentration increased from 8.34 ± 0.28 mg/kg in UOJ to 21.07 ± 0.37 mg/kg after 24 h and remained relatively stable thereafter (22.69 ± 0.97 mg/kg). In contrast, WKG fermentation caused a rapid increase to 52.71 ± 0.37 mg/kg after 24 h, followed by a significant decline to 28.70 ± 2.53 mg/kg after 48 h, suggesting that sodium was released primarily during the early stages of fermentation. The GLM for this mineral (Table S2) was highly significant (R^2^ = 0.994; adjusted R^2^ = 0.993; F = 1038.83, *p* < 0.001; ηp^2^ = 0.994). Significant effects of kefir grain type (F = 1382.10, *p* < 0.001, ηp^2^ = 0.982), fermentation time (F = 488.98, *p* < 0.001, ηp^2^ = 0.951), and their interaction (F = 640.18, *p* < 0.001, ηp^2^ = 0.962) were observed. These results corroborate the markedly different temporal patterns of Na concentration between MKG and WKG fermentations (Figure 6).

The trace elements exhibited greater variability. Iron and zinc generally decreased after fermentation, with concentrations falling below 0.2 mg/kg in some samples, suggesting possible microbial uptake. Copper exhibited a slight increase during MKG fermentation and a decrease during WKG fermentation. Selenium showed only minor fluctuations, decreasing to below 0.2 mg/kg after 24 h of MKG fermentation before returning to concentrations comparable to those of UOJ after 48 h. In WKG-fermented samples, Se increased after 24 h but decreased after prolonged incubation.

The GLM for Fe concentration (Table S2) was highly significant (F = 1024.64, *p* < 0.001, ηp^2^ = 0.994), with significant main effects of KG-TYPE (F = 140.68, *p* < 0.001, ηp^2^ = 0.849) and F-TIME (F = 131.73, *p* < 0.001, ηp^2^ = 0.840), as well as a significant KG-TYPE × F-TIME interaction (F = 45.16, *p* < 0.001, ηp^2^ = 0.644). These results indicate substantial differences in Fe concentration among KG-TYPE groups and an overall difference between 24 and 48 h, with Fe concentrations being higher at 24 h. However, the significant KG-TYPE × F-TIME interaction indicates that Fe concentration did not change in the same manner from 24 to 48 h in MKG- and WKG-fermented samples (Figure 6).

For zinc, the GLM (Table S2) explained a very large proportion of the variability in Zn concentration (F = 96.82, *p* < 0.001, ηp^2^ = 0.939). However, the effect of KG-TYPE was not statistically significant (F = 4.21, *p* = 0.051, ηp^2^ = 0.144), although it was close to the conventional significance threshold. Thus, after accounting for F-TIME and the interaction, there was insufficient evidence at the 5% significance level to conclude that Zn concentrations differed overall among KG-TYPE groups. Nevertheless, the effect size was moderate-to-large (ηp^2^ = 0.144), suggesting that KG-TYPE may have a meaningful effect, although the evidence was insufficient to establish statistical significance under the conventional α = 0.05 criterion. F-TIME had a very strong effect (F = 337.96, *p* < 0.001, ηp^2^ = 0.931), and the KG-TYPE × F-TIME interaction was statistically significant (F = 5.86, *p* = 0.023, ηp^2^ = 0.190). This indicates that the effect of fermentation time on Zn concentration depended on KG-TYPE, or equivalently, that differences among KG-TYPE groups changed according to fermentation time (Figure 6).

Copper concentration (Table S2) was significantly affected by KG-TYPE (F = 5.09, *p* = 0.033, ηp^2^ = 0.169) and by the KG-TYPE × F-TIME interaction (F = 8.40, *p* = 0.008, ηp^2^ = 0.252). However, the F-TIME main effect was not significant (F = 3.50, *p* = 0.073, ηp^2^ = 0.123). Therefore, Cu concentrations differed among grain types, but the response to fermentation duration depended on the grain type. The significant interaction is particularly relevant because it indicates that the effect of extending fermentation from 24 to 48 h was not uniform between MKG and WKG (Figure 6).

For selenium (Table S2), the results revealed significant effects of KG-TYPE (F = 203.73, *p* < 0.001, ηp^2^ = 0.891), F-TIME (F = 148.53, *p* < 0.001, ηp^2^ = 0.856), and their interaction (F = 288.02, *p* < 0.001, ηp^2^ = 0.920). These results confirmed that the effect of fermentation time on Se concentration depended strongly on KG-TYPE (Figure 6).

Overall, the observed changes in mineral composition likely resulted from the combined effects of microbial metabolism and the intrinsic composition of the kefir grains. Increases in mineral concentrations may reflect release from the grains, including components retained from the activation media, and microbial cell lysis [22,83]. Fermentation has been reported to degrade mineral-binding compounds, such as phytates, tannins, and saponins [84,85], which could potentially affect mineral bioavailability; however, bioavailability was not assessed in this study, and this hypothesis requires further investigation. Conversely, decreases may be consistent with microbial assimilation during growth [83]. The distinct mineral profiles of MKG- and WKG-fermented beverages further highlight the influence of the kefir microbiota on the nutritional characteristics of fermented orange juice.

Part of the increase in mineral concentrations may be attributable to the release of components retained within the grain matrix from the activation media, such as calcium from milk in MKG or sodium from the NaCl-supplemented sucrose solution in WKG. Because neither a final-wash analysis nor a washed-grain control incubated without substrate was performed, the relative contributions of activation-medium carryover and grain-derived release to the observed mineral changes cannot be quantified. Future studies should include such controls.

### 3.5. Changes in the Volatile Composition of Orange Juice During Kefir Fermentation

**Figure 7:**
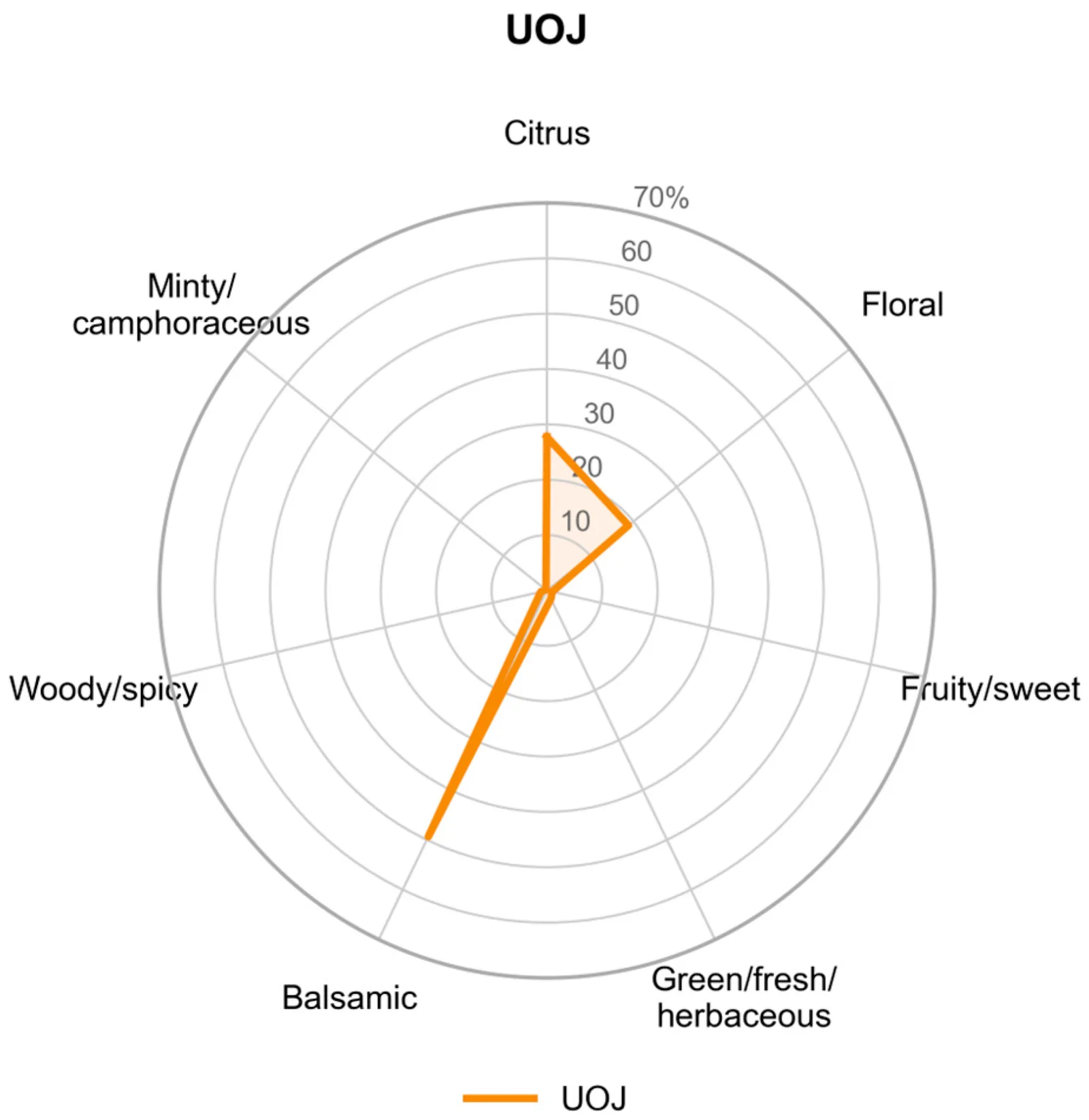
Spider plot of the percentage contribution of each aroma category (Table S4) to the aroma profile of unfermented orange juice (UOJ).

**Figure 8:**
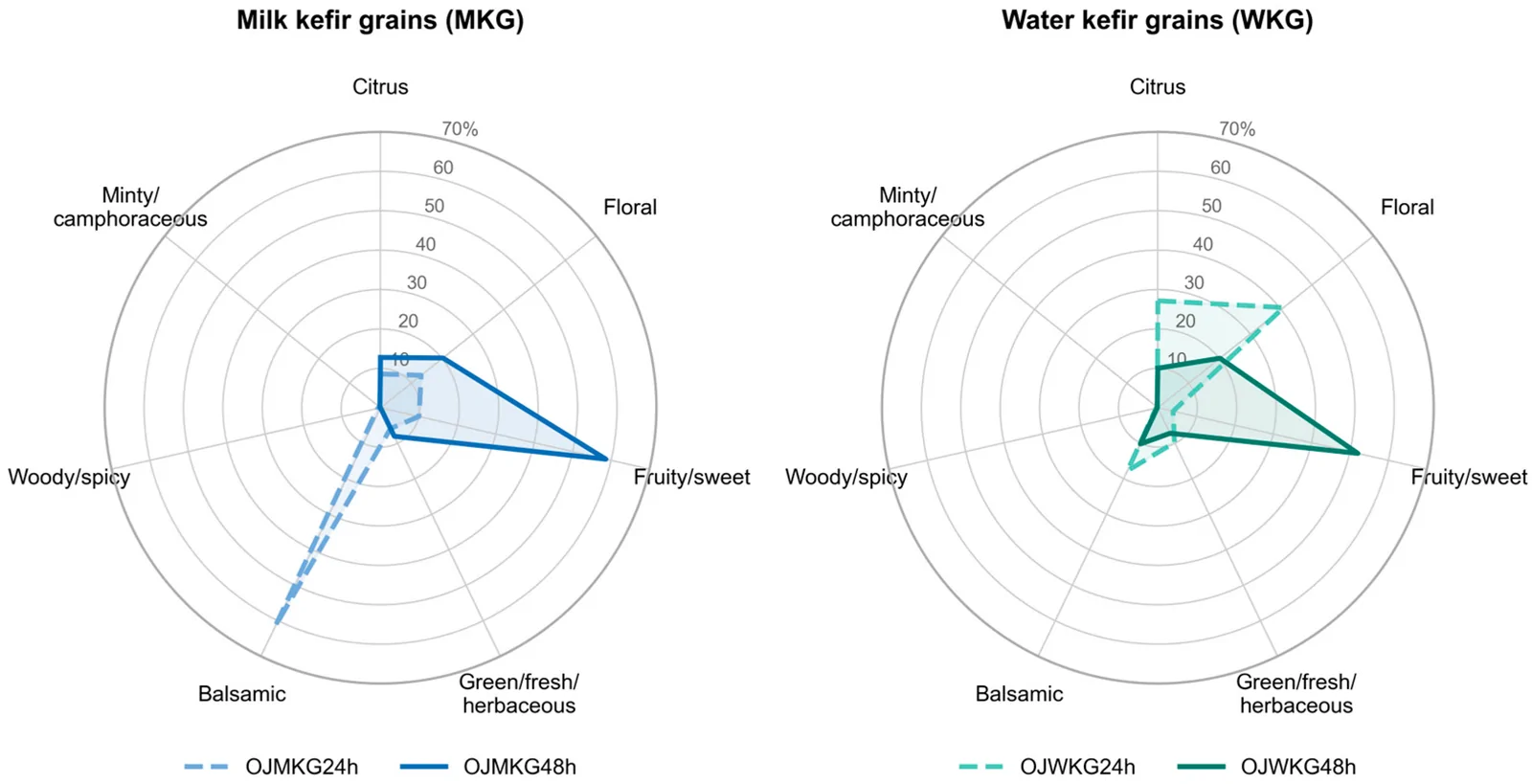
Spider plots of the percentage contribution of each aroma category (Table S4) to the aroma profiles of orange juice fermented with milk kefir grains (OJMKG24h and OJMKG48h) and water kefir grains (OJWKG24h and OJWKG48h) for 24 and 48 h.

Fermentation with milk kefir grains (MKG) and water kefir grains (WKG) markedly altered the volatile organic compound (VOC) profile of orange juice, as reflected by changes in VOC concentrations (Table 1), odor activity values (OAVs; Table 2), and the relative contribution of aroma-active VOCs (OAV ≥ 1) to seven aroma categories (Table S4; Figure 7 and Figure 8). For this analysis, VOCs were classified into citrus, floral, fruity/sweet, green/fresh/herbaceous, balsamic, woody/spicy, and minty/camphoraceous categories according to their dominant aroma descriptors (Table 2).

Thirty-eight VOCs belonging to the alcohol, aldehyde, organic acid, ester, ketone, and terpenoid classes were identified. Overall, fermentation promoted the formation of aroma-active alcohols, esters, and oxygenated terpenoids while reducing characteristic citrus terpenes and aldehydes, leading to distinct aroma profiles that depended on kefir grain type and fermentation time.

Terpenoids were the predominant VOC class in unfermented orange juice (UOJ), with D-limonene (136.89 ± 1.33 mg/L) as the major compound, followed by β-myrcene (16.27 ± 1.27 mg/L), γ-terpinene (3.86 ± 0.00 mg/L), α-pinene (1.94 ± 0.09 mg/L), caryophyllene (1.83 ± 0.00 mg/L), sabinene (1.30 ± 0.00 mg/L), and β-ocimene (1.29 ± 0.00 mg/L) (Table 1). Fermentation substantially reshaped this profile, indicating extensive microbial biotransformation of citrus monoterpenes. D-Limonene showed the greatest decline, decreasing to 83.20 ± 0.28 and 23.31 ± 2.05 mg/L after 24 and 48 h of MKG fermentation, respectively. During WKG fermentation, its concentration dropped rapidly within the first 24 h (43.36 ± 0.59 mg/L) and then remained relatively stable (43.05 ± 3.21 mg/L). Similar decreases were observed for β-myrcene, γ-terpinene, α-pinene, β-ocimene, sabinene, α-copaene, caryophyllene, α-humulene, and farnesene, suggesting microbial conversion of terpene hydrocarbons into oxygenated derivatives [86,87]. This depletion was accompanied by the accumulation of oxygenated monoterpenes, particularly geraniol and β-citronellol, together with dynamic changes in linalool, terpinen-4-ol, α-terpineol, trans-carveol, and δ-carvone. These observations agree with the terpene interconversion pathways proposed by Xie et al. [86], in which limonene and linalool act as central intermediates undergoing oxidation, reduction, hydration, and isomerization [86,87,88]. Overall, MKG promoted a more extensive conversion of terpene hydrocarbons than WKG, especially after 48 h, whereas WKG retained higher concentrations of limonene and α-terpineol.

The GLM analysis confirmed pronounced treatment- and time-dependent changes in terpenoids (*p* ≤ 0.001), particularly for D-limonene, β-myrcene, α-pinene, α-copaene, caryophyllene, 1,3,8-p-menthatriene, α-humulene, and sabinene (Table S3). For most compounds, the largest effects were associated with the KG-TYPE × F-TIME interaction (*p* < 0.010), indicating that temporal changes depended strongly on the kefir grain type. In contrast, β-ocimene showed significant effects of KG-TYPE and the interaction (*p* = 0.014), but not F-TIME alone (*p* = 0.090), whereas γ-terpinene was affected primarily by fermentation time (*p* < 0.010), with no significant effects of KG-TYPE (*p* = 0.594) or the interaction (*p* = 0.430). Farnesene was excluded from the GLM because no within-treatment variation was observed.

Alcohols showed the largest increase in absolute concentration, rising from 42.70 ± 0.63 mg/L in UOJ to 64.93 ± 0.87 and 61.63 ± 0.82 mg/L after 48 h of MKG and WKG fermentation, respectively (Table 1). This increase mainly reflects yeast metabolism through the Ehrlich pathway together with microbial terpene biotransformation [18]. Among the higher alcohols, 1-octanol, 1-hexanol, (Z)-3-hexen-1-ol, and 3-methyl-1-butanol increased markedly, whereas 2-phenylethanol was produced only during fermentation. In contrast, linalool, α-terpineol, and nerol declined, while geraniol and β-citronellol accumulated, further supporting continuous oxygenated terpene interconversion. Consequently, the alcohol fraction became enriched in floral, herbaceous, and fruity aroma compounds (Table 2).

The GLM analysis showed that KG-TYPE, F-TIME, and their interaction significantly affected most alcohols, although the response patterns differed among compounds (Table S3). Three main trends were identified. First, KG-TYPE, F-TIME, and their interaction were all significant (*p* < 0.050) for most alcohols, including 1-octanol, (Z)-3-hexen-1-ol, linalool, 3-methyl-1-butanol, β-citronellol, terpinen-4-ol, α-terpineol, nerol, and 2-phenylethanol. Second, geraniol was significantly affected by KG-TYPE and the interaction (*p* < 0.002), whereas F-TIME alone was not significant (*p* = 0.176). Third, 1-hexanol and trans-carveol were influenced only by fermentation time (*p* < 0.003), with no significant effects of KG-TYPE or the interaction (*p* > 0.050). Overall, fermentation time was the principal factor driving changes in alcohol concentrations, whereas kefir grain type further modulated these responses.

Esters exhibited the largest relative increase among all VOC classes, with total concentrations rising from 0.87 ± 0.04 mg/L in UOJ to 14.44 ± 0.62 mg/L after 48 h of MKG fermentation and 12.57 ± 0.59 mg/L after 48 h of WKG fermentation (Table 1). Ethyl butanoate was the predominant fermentation-derived ester, followed by ethyl hexanoate and ethyl octanoate. Their accumulation reflects yeast alcohol acetyltransferase activity, which catalyzes esterification between alcohols and short- or medium-chain fatty acids [89,90]. Owing to their very low odor thresholds, these esters became major contributors to the fruity and sweet aroma of fermented orange juice, particularly after 48 h. Consequently, the fruity/sweet aroma category increased markedly, accounting for 58.6% and 52.0% of the total ΣOAV in OJMKG48h and OJWKG48h, respectively (Table S4; Figure 8).

The four esters displayed consistent but not identical GLM responses (Table S3). Both KG-TYPE and F-TIME significantly affected all compounds (*p* < 0.001), whereas the KG-TYPE × F-TIME interaction was significant for ethyl hexanoate, ethyl octanoate, and octyl acetate (*p* < 0.001), but not for ethyl butanoate (*p* = 0.452). Ethyl butanoate was predominantly time-dependent (F = 633.45, *p* < 0.001, ηp^2^ = 0.962), showing a marked increase between 24 and 48 h irrespective of kefir grain type. Ethyl hexanoate exhibited a strong grain type-dependent temporal response, with an exceptionally large interaction effect (F = 537.64, *p* < 0.001, ηp^2^ = 0.956), indicating distinct temporal patterns in MKG and WKG. Ethyl octanoate and octyl acetate also showed significant interactions, demonstrating that their evolution during fermentation depended strongly on kefir grain type. Ethyl octanoate increased after 24 h but declined following prolonged fermentation, whereas octyl acetate increased progressively over time.

Aldehydes declined sharply during fermentation, with total concentrations decreasing from 28.26 ± 1.15 mg/L in UOJ to approximately 1.24 mg/L in MKG-fermented beverages and from 3.35 ± 0.06 to 0.70 ± 0.03 mg/L in WKG-fermented beverages (Table 1). Decanal, dodecanal, neral, and geranial were almost completely depleted, whereas hexanal remained relatively stable and increased after prolonged MKG fermentation, possibly owing to continued lipid oxidation. This depletion of citrus aldehydes is consistent with microbial alcohol dehydrogenase activity, which converts aldehydes into their corresponding alcohols [86,91,92].

The GLM analysis indicated that all five aldehydes were significantly affected by KG-TYPE, F-TIME, and their interaction (*p* < 0.001), although the magnitude of these effects varied among compounds (Table S3). Decanal and dodecanal showed rapid declines throughout fermentation, with particularly pronounced reductions in MKG. Neral and geranial followed a similar pattern and were no longer detected after 48 h in either treatment. In contrast, hexanal exhibited distinct temporal responses between kefir grain types: its concentration increased markedly during MKG fermentation but remained essentially unchanged in WKG. This difference produced a highly significant KG-TYPE × F-TIME interaction with exceptionally large effect sizes, indicating that hexanal accumulation was specific to MKG fermentation.

Volatile organic acids were detected only in MKG-fermented samples, where octanoic, n-decanoic, and dodecanoic acids accumulated progressively. These medium-chain fatty acids are typical products of yeast lipid metabolism and serve as precursors for ethyl ester synthesis [93,94,95]. Their absence in WKG samples suggests either rapid conversion into esters or concentrations below the detection limit.

The GLM (Table S3) confirmed highly significant effects of KG-TYPE, F-TIME, and their interaction for all three organic acids (*p* < 0.001), although the magnitude of the response differed among compounds. Octanoic and dodecanoic acids were detected only after 48 h of MKG fermentation, whereas n-decanoic acid showed the strongest quantitative response, increasing more than tenfold between 24 and 48 h. In contrast, none of these acids was detected in WKG-fermented samples. The consistently strong interaction effects indicate that the influence of fermentation time depended entirely on kefir grain type, with acid accumulation occurring exclusively in MKG. These findings provide strong evidence that MKG favored the production or accumulation of medium-chain fatty acids during the later stages of fermentation, whereas WKG did not.

Ketone concentrations increased moderately during fermentation, from 0.87 ± 0.04 mg/L in the unfermented juice to 0.96 ± 0.03 mg/L at 24 h and 2.23 ± 0.10 mg/L at 48 h in the MKG fermentation, and to 1.83 ± 0.09 mg/L at 24 h and 1.75 ± 0.06 mg/L at 48 h in the WKG fermentation. The formation of 3-octanone together with the accumulation of δ-carvone and nootkatone suggests oxidative transformation of terpene and lipid precursors, whereas the concurrent depletion of limonene further supports microbial terpene biotransformation [86,96,97].

The three ketones differed in their response to the experimental factors (Table S3). 3-Octanone was absent from UOJ but increased steadily during fermentation in both treatments, with significant effects of KG-TYPE, F-TIME, and their interaction (*p* < 0.001). δ-Carvone remained comparatively stable, showing only a slight increase over time, although all three GLM effects were significant. Nootkatone exhibited contrasting temporal patterns between treatments, decreasing initially and then increasing markedly in MKG, whereas higher concentrations were maintained throughout WKG fermentation. The GLM confirmed significant effects of KG-TYPE, F-TIME, and their interaction (*p* < 0.001). Overall, 3-octanone and nootkatone displayed the strongest responses to fermentation, whereas δ-carvone showed more moderate changes. The significant interaction terms for all three compounds indicate that the effect of fermentation time depended on kefir grain type.

The compositional changes in VOCs (Table 1) were reflected in the odor activity values (Table 2) and in the distribution of aroma categories (Table S4; Figure 7 and Figure 8).

However, the aroma categories inferred from the VOC profiles and OAVs represent potential aroma characteristics and should not be interpreted as evidence of actual human sensory perception. As no human sensory panel evaluation was performed in the present study, the relationship between the instrumental VOC/OAV results and actual sensory perception remains to be established.

Although the concentrations of D-limonene and β-myrcene decreased markedly during fermentation, both compounds remained important contributors to the aroma profile because of their high OAVs. At the same time, fermentation enhanced the relative contribution of highly odor-active esters, higher alcohols, and oxygenated terpenoids, particularly ethyl butanoate, ethyl hexanoate, 2-phenylethanol, geraniol, and β-citronellol. As a result, the OAV-based aroma profile shifted from one dominated by balsamic (β-myrcene, 49.4%), followed by citrus (27.8%) and floral (18.9%) notes in unfermented orange juice (Figure 7) to one characterized by an increasing contribution of fruity/sweet compounds in the fermented beverages, particularly after 48 h (Figure 8).

The spider plots (Figure 7 and Figure 8) illustrate the progressive evolution of the aroma profile throughout fermentation. The marked increase in fruity/sweet notes, particularly after 48 h, reflects the accumulation of ethyl esters, whereas the floral contribution is associated with the production of geraniol, β-citronellol, and 2-phenylethanol, together with the persistence of highly odor-active compounds such as linalool and terpinen-4-ol. Although both kefir grain types followed the same overall trend, the depletion of β-myrcene occurred earlier with WKG, and WKG beverages showed a markedly higher citrus and floral contribution after 24 h (27.2% and 40.8%, vs. 8.6% and 13.2% for MKG), whereas the MKG beverage was still dominated by the balsamic category (61.4%). After 48 h, both beverages showed similar citrus (12.8% and 10.0% for MKG and WKG, respectively) and floral (20.3% and 20.1%) contributions, whereas MKG showed a slightly higher fruity/sweet contribution (58.6% vs. 52.0%), consistent with its greater depletion of terpene hydrocarbons and higher production of esters.

Kefir fermentation shifted the OAV-based aroma profile from balsamic and citrus contributions toward fruity/sweet notes, with the extent of the shift depending on grain type and fermentation time. Sensory analysis is needed to establish whether these instrumental changes are perceptible to consumers.

**Table 2:** Aroma descriptors, odor thresholds (OT, mg/L), and odor activity values (OAVs) of aroma-active volatile organic compounds (VOCs; OAV ≥ 1.0 in at least one sample) in unfermented orange juice (UOJ) and orange juice fermented with milk kefir grains (OJMKG24h and OJMKG48h) or water kefir grains (OJWKG24h and OJWKG48h) for 24 and 48 h.

			OAVs					
No.	Compound	Aroma Descriptor	* OT	UOJ	OJMKG24h	OJMKG48h	OJWKG24h	OJWKG48h
Alcohols								
1	1-Octanol	Herbal [98]	0.1258	45.09	81.56	146.41	46.17	142.86
2	1-Hexanol	Grass, floral, resinous, paper-like [96]	0.0056	270.10	1053.35	1185.34	1000.00	1146.21
3	(Z)-3-Hexen-1-ol	Fatty, grassy, leafy [96]	0.07	2.20	4.18	8.62	3.18	6.28
4	Linalool	Floral [96,99], green [96], fruity, sweet [99]	0.006	1644.04	1464.46	1054.60	1870.42	1017.04
5	3-Methyl-1-butanol	Fusel, oily, alcoholic, whiskey-like, fruity, banana [100]	0.0159	105.19	271.96	587.33	139.76	200.62
6	β-Citronellol	Sweet, floral, rose-like [101]	1.131	1.09	1.88	2.78	1.63	2.06
7	Geraniol	Fruity, citrusy, rose-like [96]	0.0025	471.95	1039.46	1773.34	2248.00	1266.67
8	Terpinen-4-ol	Woody, floral [96]	0.0037 ^a^	3070.96	1222.57	2859.16	2853.49	2891.88
9	α-Terpineol	Lemon-like, minty, piney [99], green, violet [96]	1.2	6.10	5.40	-	5.67	5.39
10	*trans*-Carveol	Caraway-like, green, oily [102]	0.25	7.56	7.73	3.75	7.65	4.08
11	2-Phenylethanol	Rose-like [103]	0.56423	-	3.87	7.99	3.56	6.77
Aldehydes								
12	Decanal	Green, citrus-like [96,99], fatty [99]	0.003	6310.85	83.76	-	407.51	94.75
13	Dodecanal	Soapy, waxy, aldehydic, citrusy, green, floral [104]	0.01	441.35	39.78	20.11	86.88	-
14	Hexanal	Grassy [96,99], green, soapy [99]	0.005	92.51	54.45	208.43	83.95	84.51
15	Neral	Citrus, lemon, minty [99]	0.053	29.33	2.20	-	5.26	-
16	Geranial	Citrusy [96,99], minty, green [99], floral [96]	0.032	90.70	6.52	-	17.54	-
Esters								
17	Ethyl butanoate	Pineapple-like, fruity [96,99]	0.0009	170.62	1764.31	10,843.31	254.22	9901.42
18	Ethyl hexanoate	Orange-like, fruity [96]	0.005	28.65	240.77	607.58	319.98	380.11
19	Ethyl octanoate	Fruity [96]	0.0193	-	44.73	34.14	63.35	47.85
20	Octyl acetate	Fruity, floral [103]	0.047	12.15	18.94	20.94	12.95	17.73
Ketones								
21	3-Octanone	Stale, moldy, slightly fruity, sweet, pear-like, candy-like, cooked [105]	0.0214	-	6.07	14.02	11.68	15.88
22	δ-Carvone	Minty, caraway-like [96]	0.16	1.52	2.08	2.44	1.49	2.20
23	Nootkatone	Grapefruit-like, green [99], pungent, aromatic, musty [96]	0.28	2.22	1.79	5.51	4.79	3.79
Terpenoids								
24	D-Limonene	Citrus [99], lemon-like, minty [96,99]	0.2	684.44	416.01	116.54	216.79	215.24
25	β-Myrcene	Geranium-like [99], mossy, musty, balsamic [96,99]	0.0012	13,555.75	12,800.24	-	2099.81	1991.88
26	α-Pinene	Pine, citrus peel, woody [96]	0.014	138.42	124.75	15.08	-	-
27	β-Ocimene	Green, spicy [106,107], citrusy, tropical, terpene-like, woody [102]	0.034	37.94	16.84	16.14	12.64	16.15
28	γ-Terpinene	Sweet, citrusy [99]	1.0	3.86	0.90	0.71	0.89	0.76
29	α-Copaene	Vegetable-like, hop-like, woody [100]	0.006 ^b^	144.44	26.50	-	41.71	27.23
30	Caryophyllene	Spicy, woody [100]	0.064	28.52	2.23	-	14.21	7.17
31	1,3,8-p-Menthatriene	Turpentine-like, camphoraceous, herbal, woody [102]	0.015	49.32	41.48	40.74	7.20	38.52
32	α-Humulene	Citrusy, herbal [106], floral, woody [108]	0.16	1.95	-	-	0.64	-
33	Sabinene	Woody [101], green [103]	0.98	1.33	0.77	0.60	0.63	0.57
34	Farnesene	Sweet, floral [103]	0.087	5.26	-	-	-	-

* Odor thresholds (OTs) were obtained from van Gemert [109], unless otherwise indicated. ^a^ Pan et al. [96], ^b^ Iwasa et al. [110].

## 4. Conclusions

This study demonstrates that both milk kefir grains (MKG) and water kefir grains (WKG) are effective starter cultures for orange juice fermentation, producing beverages with distinct physicochemical, microbiological, nutritional, and aromatic characteristics. Fermentation promoted sugar utilization and the production of organic acids, ethanol, glycerol, and antimicrobial metabolites, while increasing free microbial biomass and kefir grain weight.

The ethanol content, particularly in the WKG beverage fermented for 48 h (1.37% *v*/*v*), is a key quality attribute that will determine beverage classification, labeling, and target consumers, and must be controlled in future product development.

Both kefir consortia adapted rapidly to the orange juice matrix, reaching high viable counts of lactic acid bacteria (6–7 log CFU/mL), acetic acid bacteria (5–6 log CFU/mL), and yeasts (approximately 8 log CFU/mL). Although selective-media counts confirmed the presence of viable microbial groups, they do not establish probiotic status. Further studies should assess strain identity, probiotic properties, gastrointestinal tolerance, adhesion, safety, antibiotic resistance, and in vivo health effects to determine the beverages’ probiotic potential.

Kefir fermentation also proved to be an effective strategy for modifying the volatile profile of orange juice through extensive microbial biotransformation. Both MKG and WKG increased the OAV-based contribution of fruity/sweet compounds by increasing the formation of aroma-active esters, higher alcohols, and oxygenated terpenoids while reducing citrus aldehydes and terpene hydrocarbons. However, the extent of these changes depended on the kefir grain type. MKG produced a more pronounced transformation of the aroma profile than WKG, indicating that the kefir consortium and fermentation time can be selected to modify the volatile composition of fermented orange juice, although the sensory relevance of these changes remains to be confirmed.

Together, the results identify grain type and fermentation time as key determinants of beverage composition and provide a basis for optimizing non-dairy kefir production. Since each grain type was tested in a single fermentation run, reproducibility across batches should be established. Further studies should examine consumer acceptance, storage stability, nutrient bioavailability, and in vivo health effects to better establish the commercial potential of these fermented beverages.

## Figures and Tables

**Table 1:** Concentrations (mg/L, mean ± standard deviation) of volatile organic compounds (VOCs) in orange juice before fermentation (UOJ) and after fermentation with milk kefir grains (OJMKG24h and OJMKG48h) or water kefir grains (OJWKG24h and OJWKG48h) for 24 and 48 h.

No.	Compound	UOJ	OJMKG24h	OJMKG48h	OJWKG24h	OJWKG48h
Alcohols						
1	1-Octanol	5.67 ± 0.01	10.26 ± 0.00	18.42 ± 0.09	5.81 ± 0.31	17.97 ± 0.38
2	1-Hexanol	1.51 ± 0.00	5.90 ± 0.56	6.64 ± 0.98	5.60 ± 0.02	6.42 ± 0.50
3	(Z)-3-Hexen-1-ol	0.15 ± 0.00	0.29 ± 0.00	0.60 ± 0.01	0.22 ± 0.00	0.44 ± 0.03
4	Linalool	9.86 ± 0.03	8.79 ± 1.95	6.33 ± 0.08	11.22 ± 0.74	6.10 ± 1.40
5	3-Methyl-1-butanol	1.67 ± 0.01	4.32 ± 0.04	9.34 ± 0.53	2.22 ± 0.03	3.19 ± 0.02
6	β-Citronellol	1.23 ± 0.01	2.12 ± 0.01	3.14 ± 0.05	1.84 ± 0.02	2.33 ± 0.01
7	Geraniol	1.18 ± 0.00	2.60 ± 0.42	4.43 ± 0.51	5.62 ± 0.23	3.17 ± 0.99
8	Terpinen-4-ol	11.36 ± 0.02	4.52 ± 0.00	10.58 ± 1.27	10.56 ± 0.03	10.70 ± 1.14
9	α-Terpineol	7.32 ± 0.01	6.47 ± 0.00	N.D.	6.80 ± 0.29	6.47 ± 0.45
10	trans-Carveol	1.89 ± 0.04	1.93 ± 0.21	0.94 ± 0.03	1.91 ± 0.01	1.02 ± 0.02
11	Nerol	0.84 ± 0.02	N.D.	N.D.	0.48 ± 0.14	N.D.
12	2-Phenylethanol	N.D.	2.18 ± 0.01	4.51 ± 0.30	2.01 ± 0.03	3.82 ± 0.05
	No. of Alcohols	11	11	10	12	11
	Total concentrations	42.70 ± 0.63	49.40 ± 0.53	64.93 ± 0.87	54.30 ± 0.60	61.63 ± 0.82
Aldehydes						
13	Decanal	18.93 ± 0.23	0.25 ± 0.09	N.D.	1.22 ± 0.17	0.28 ± 0.00
14	Dodecanal	4.41 ± 0.11	0.40 ± 0.10	0.20 ± 0.06	0.87 ± 0.03	N.D.
15	Hexanal	0.46 ± 0.01	0.27 ± 0.00	1.04 ± 0.02	0.42 ± 0.03	0.42 ± 0.01
16	Neral	1.55 ± 0.03	0.12 ± 0.00	N.D.	0.28 ± 0.03	N.D.
17	Geranial	2.90 ± 0.01	0.21 ± 0.07	N.D.	0.56 ± 0.01	N.D.
	No. of Aldehydes	5	5	2	5	2
	Total concentrations	28.26 ± 1.15	1.24 ± 0.02	1.24 ± 0.07	3.35 ± 0.06	0.70 ± 0.03
Organic acids						
18	Octanoic acid	N.D.	N.D.	0.26 ± 0.02	N.D.	N.D.
19	Dodecanoic acid	N.D.	N.D.	0.23 ± 0.01	N.D.	N.D.
20	n-Decanoic acid	N.D.	0.32 ± 0.00	3.67 ± 0.07	N.D.	N.D.
	No. of Organic Acids	N.D.	1	3	0	0
	Total concentrations	0	0.32 ± 0.00	4.16 ± 0.28	N.D.	N.D.
Esters						
21	Ethyl butanoate	0.15 ± 0.00	1.59 ± 0.02	9.76 ± 0.04	0.23 ± 0.01	8.91 ± 1.83
22	Ethyl hexanoate	0.14 ± 0.01	1.20 ± 0.18	3.04 ± 0.04	1.60 ± 0.01	1.90 ± 0.00
23	Ethyl octanoate	N.D.	0.86 ± 0.01	0.66 ± 0.03	1.22 ± 0.02	0.92 ± 0.02
24	Octyl acetate	0.57 ± 0.01	0.89 ± 0.02	0.98 ± 0.01	0.61 ± 0.04	0.83 ± 0.02
	No. of Esters	3	4	4	4	4
	Total concentrations	0.87 ± 0.04	4.54 ± 0.05	14.44 ± 0.62	3.66 ± 0.09	12.57 ± 0.59
Ketones						
25	3-Octanone	N.D.	0.13 ± 0.00	0.30 ± 0.01	0.25 ± 0.02	0.34 ± 0.01
26	δ-Carvone	0.24 ± 0.00	0.33 ± 0.07	0.39 ± 0.01	0.24 ± 0.02	0.35 ± 0.01
27	Nootkatone	0.62 ± 0.01	0.50 ± 0.10	1.54 ± 0.09	1.34 ± 0.09	1.06 ± 0.02
	No. of Ketones	2	3	3	3	3
	Total concentrations	0.87 ± 0.04	0.96 ± 0.03	2.23 ± 0.10	1.83 ± 0.09	1.75 ± 0.06
Terpenoids						
28	D-Limonene	136.89 ± 1.33	83.20 ± 0.28	23.31 ± 2.05	43.36 ± 0.59	43.05 ± 3.21
29	β-Myrcene	16.27 ± 1.27	15.36 ± 0.85	N.D.	2.52 ± 0.13	2.39 ± 0.32
30	α-Pinene	1.94 ± 0.09	1.75 ± 0.05	0.21 ± 0.00	N.D.	N.D.
31	β-Ocimene	1.29 ± 0.00	0.57 ± 0.05	0.55 ± 0.10	0.43 ± 0.00	0.55 ± 0.10
32	γ-Terpinene	3.86 ± 0.00	0.90 ± 0.21	0.71 ± 0.03	0.89 ± 0.01	0.76 ± 0.02
33	α-Copaene	0.87 ± 0.00	0.16 ± 0.07	N.D.	0.25 ± 0.00	0.16 ± 0.00
34	Caryophyllene	1.83 ± 0.00	0.14 ± 0.01	N.D.	0.91 ± 0.00	0.46 ± 0.01
35	1,3,8-p-Menthatriene	0.74 ± 0.00	0.62 ± 0.07	0.61 ± 0.03	0.11 ± 0.00	0.58 ± 0.05
36	α-Humulene	0.31 ± 0.00	N.D.	N.D.	0.10 ± 0.01	N.D.
37	Sabinene	1.30 ± 0.00	0.75 ± 0.02	0.59 ± 0.06	0.62 ± 0.02	0.56 ± 0.03
38	Farnesene	0.46 ± 0.00	N.D.	N.D.	N.D.	N.D.
	No. of Terpenoids	11	9	6	9	8
	Total concentrations	165.75 ± 6.51	103.46 ± 3.98	25.98 ± 1.12	49.18 ± 2.07	48.51 ± 2.06

N.D.: not detected. The effects of fermentation time, kefir grain type, and their interaction were analyzed using a general linear model, with statistical significance set at *p* < 0.05.

## Data Availability

The data supporting the findings of this study are available within the article and its Supplementary Materials. Further inquiries can be directed to the corresponding author.

## Supplementary Materials

The following supporting information can be downloaded at: https://www.mdpi.com/article/10.3390/foods15193544/s1, Table S1: Summary of the general linear model assessing the effects of kefir grain type (KG-TYPE), fermentation time (F-TIME), and their interaction (KG-TYPE × F-TIME) on vitamin concentrations in unfermented juice and in samples fermented with milk kefir grains (MKG) or water kefir grains (WKG) for 24 or 48 h. df: degrees of freedom, ηp^2^: partial eta-squared, represents an estimate of the effect size for the corresponding source. Table S2: Summary of the general linear model assessing the effects of kefir grain type (KG-TYPE), fermentation time (F-TIME), and their interaction (KG-TYPE × F-TIME) on mineral concentrations in unfermented juice and in samples fermented with milk kefir grains (MKG) or water kefir grains (WKG) for 24 or 48 h. df: degrees of freedom, ηp^2^: partial eta-squared, represents an estimate of the effect size for the corresponding source. Table S3: Summary of the general linear model assessing the effects of kefir grain type (KG-TYPE), fermentation time (F-TIME), and their interaction (KG-TYPE × F-TIME) on volatile compound concentrations in unfermented juice and in samples fermented with milk kefir grains (MKG) or water kefir grains (WKG) for 24 or 48 h. df: degrees of freedom, ηp^2^: partial eta-squared, represents an estimate of the effect size for the corresponding source. Table S4: Classification of volatile organic compounds (VOCs) into aroma categories and the corresponding sums of odor activity values (ΣOAVs).
