# Supplementary material for: Orange Juice as a Substrate for Milk and Water Kefir Grains: Production and Characterization of Novel Kefir-like Beverages
Source: Foods. 2026 Oct 4;15(19):3544. doi: 10.3390/foods15193544 (PMC13664867; doi:10.3390/foods15193544)
Supplement: Supplementary file 1 [file foods-15-03544-s001.zip › foods-4490185-supplementary.pdf]

Supplementary Material

Table S1. Summary of the general linear model assessing the effects of kefir grain type (KG-TYPE), fermentation time (F-TIME), and their interaction (KG-TYPE × F-TIME) on vitamin concentrations in unfermented juice and in samples fermented with milk kefir grains (MKG) or water kefir grains (WKG) for 24 or 48 h. df: degrees of freedom, ηp²: partial eta-squared, represents an estimate of the effect size for the corresponding source.

**Vitamin B1**						
**Tests of Between-Subjects Effects**						
**Source**	**Type III Sum of Squares**	**df**	**Mean Square**	**F**	**Sig.**	**ηp²**
**Corrected Model**	8955.88*	4	2238.97	494.27	0.000	0.988
**Intercept**	19259.34	1	19259.34	4251.69	0.000	0.994
**KG-TYPE**	283.25	1	283.25	62.53	0.000	0.714
**F-TIME**	2393.00	1	2393.00	528.28	0.000	0.955
**KG-TYPE × F-TIME**	6.15	1	6.15	1.36	0.255	0.052
**Error**	113.24	25	4.53			
**Total**	24428.10	30				
**Corrected Total**	9069.12	29				
***R^2** = 0.988 (**Adjusted R^2** = 0.986)						
**Vitamin B2**						
**Corrected Model**	1105.55*	4	276.39	127.03	0.000	0.988
**Intercept**	5541.50	1	5541.50	2546.92	0.000	0.994
**KG-TYPE**	376.19	1	376.19	172.90	0.000	0.714
**F-TIME**	36.04	1	36.04	16.56	0.000	0.955
**KG-TYPE × F-TIME**	7.26	1	7.26	3.33	0.080	0.052
**Error**	54.39	25	2.18			
**Total**	6103.59	30				
**Corrected Total**	1159.94	29				
***R^2** = 0.953 (**Adjusted R^2** = 0.946)						
**Vitamin B3**						
**Corrected Model**	6894.84*	4	1723.71	540.99	0.000	0.989
**Intercept**	34560.56	1	34560.56	10846.70	0.000	0.998
**KG-TYPE**	1765.60	1	1765.60	554.14	0.000	0.957
**F-TIME**	2834.77	1	2834.77	889.70	0.000	0.973
**KG-TYPE × F-TIME**	2231.62	1	2231.62	700.40	0.000	0.966
**Error**	79.65	25	3.19			
**Total**	42739.43	30				
**Corrected Total**	6974.50	29				
***R^2** = 0.989 (**Adjusted R^2** = 0.987)						
**Vitamin B6 (pyridoxal)**						
**Corrected Model**	58397.28*	4	14599.32	59.91	0.000	0.906
**Intercept**	3035463.90	1	3035463.90	12455.56	0.000	0.998
**KG-TYPE**	30972.45	1	30972.45	127.09	0.000	0.836
**F-TIME**	3962.25	1	3962.25	16.26	0.000	0.394

**KG-TYPE × F-TIME**	1977.24	1	1977.24	8.11	0.009	0.245
**Error**	6092.59	25	243.70			
**Total**	3145736.78	30				
**Corrected Total**	64489.86	29				
***R**^{2} = 0.906 (**Adjusted R**^{2} = 0.890)						
**Vitamin B6 (pyridoxine)**						
**Corrected Model**	40.52*	4	10.13	66.60	0.000	0.914
**Intercept**	460.32	1	460.32	3026.65	0.000	0.992
**KG-TYPE**	1.89	1	1.89	12.46	0.002	0.333
**F-TIME**	7.96	1	7.96	52.370	0.000	0.677
**KG-TYPE × F-TIME**	29.21	1	29.21	192.089	0.000	0.885
**Error**	3.80	25	0.15			
**Total**	517.74	30				
**Corrected Total**	44.32	29				
***R**^{2} = 0.914 (**Adjusted R**^{2} = 0.900)						
**Vitamin B6 (pyridoxamine)**						
**Corrected Model**	17.27*	4	4.32	409.22	0.000	0.985
**Intercept**	65.74	1	65.74	6230.02	0.000	0.996
**KG-TYPE**	11.16	1	11.16	1057.50	0.000	0.977
**F-TIME**	1.90	1	1.90	179.97	0.000	0.878
**KG-TYPE × F-TIME**	1.64	1	1.64	155.29	0.000	0.861
**Error**	0.26	25	0.01			
**Total**	80.77	30				
**Corrected Total**	17.54	29				
***R**^{2} = 0.985 (**Adjusted R**^{2} = 0.983)						
**Vitamin C**						
**Corrected Model**	667265.41*	4	166816.35	14371.58	0.000	1.000
**Intercept**	666761.093	1	666761.09	57442.88	0.000	1.000
**KG-TYPE**	50000.263	1	50000.26	4307.63	0.000	0.994
**F-TIME**	2471.753	1	2471.75	212.95	0.000	0.895
**KG-TYPE × F-TIME**	2089.592	1	2089.59	180.02	0.000	0.878
**Error**	290.184	25	11.61			
**Total**	1096156.385	30				
**Corrected Total**	667555.595	29				
***R**^{2} = 1.000 (**Adjusted R**^{2} = 0.999)						

Table S2. Summary of the general linear model assessing the effects of kefir grain type (KG-TYPE), fermentation time (F-TIME), and their interaction (KG-TYPE × F-TIME) on mineral concentrations in unfermented juice and in samples fermented with milk kefir grains (MKG) or water kefir grains (WKG) for 24 or 48 h. df: degrees of freedom, ηp²: partial eta-squared, represents an estimate of the effect size for the corresponding source.

**Potassium**						
**Tests of Between-Subjects Effects**						
**Source**	**Type III Sum of Squares**	**df**	**Mean Square**	**F**	**Sig.**	**ηp²**
**Corrected Model**	144308.73*	4	36077.18	6.69	0.001	0.517
**Intercept**	77169533.66	1	77169533.66	14321.03	0.000	0.998
**KG-TYPE**	95486.72	1	95486.72	17.72	0.000	0.415
**F-TIME**	5375.73	1	5375.73	1.00	0.327	0.038
**KG-TYPE × F-TIME**	12495.78	1	12495.78	2.32	0.140	0.085
**Error**	134713.62	25	5388.5485			
**Total**	82469765.96	30				
**Corrected Total**	279022.35	29				
***R^2** = 0.517 (**Adjusted R^2** = 0.440)						
**Phosphorus**						
**Corrected Model**	1515.25*	4	378.81	31.31	0.000	0.834
**Intercept**	860443.70	1	860443.70	71130.57	0.000	1.000
**KG-TYPE**	261.88	1	261.88	21.65	0.000	0.464
**F-TIME**	426.54	1	426.54	35.26	0.000	0.585
**KG-TYPE × F-TIME**	17.68	1	17.68	1.46	0.238	0.055
**Error**	302.42	25	12.10			
**Total**	922699.29	30				
**Corrected Total**	1817.66	29				
***R^2** = 0.834 (**Adjusted R^2** = 0.807)						
**Magnesium**						
**Corrected Model**	569.82*	4	142.46	7.30	0.000	0.539
**Intercept**	406032.20	1	406032.20	20820.46	0.000	0.999
**KG-TYPE**	207.80	1	207.80	10.66	0.003	0.299
**F-TIME**	51.77	1	51.77	2.65	0.116	0.096
**KG-TYPE × F-TIME**	9.70	1	9.70	0.50	0.487	0.020
**Error**	487.54	25	19.50			
**Total**	434926.02	30				
**Corrected Total**	1057.36	29				
***R^2** = 0.539 (**Adjusted R^2** = 0.465)						
**Calcium**						
**Corrected Model**	6621.68*	4	1655.42	353.07	0.000	0.983
**Intercept**	161215.73	1	161215.73	34384.64	0.000	0.999
**KG-TYPE**	5248.84	1	5248.84	1119.49	0.000	0.978
**F-TIME**	53.19	1	53.19	11.34	0.002	0.312
**KG-TYPE × F-TIME**	12.23	1	12.23	2.61	0.119	0.094

**Error**	117.21	25	4.69			
**Total**	183971.09	30				
**Corrected Total**	6738.89	29				
***R^2** = 0.983 (**Adjusted R^2** = 0.980)						
**Sodium**						
**Corrected Model**	6394.18*	4	1598.54	1038.83	0.000	0.994
**Intercept**	17120.94	1	17120.94	11126.17	0.000	0.998
**KG-TYPE**	2126.77	1	2126.77	1382.10	0.000	0.982
**F-TIME**	752.43	1	752.43	488.97	0.000	0.951
**KG-TYPE × F-TIME**	985.11	1	985.11	640.18	0.000	0.962
**Error**	38.47	25	1.54			
**Total**	27823.19	30				
**Corrected Total**	6432.65	29				
***R^2** = 0.994 (**Adjusted R^2** = 0.993)						
**Iron**						
**Corrected Model**	0.37*	4	0.09	1024.64	0.000	0.994
**Intercept**	2.88	1	2.88	32259.17	0.000	0.999
**KG-TYPE**	0.01	1	0.01	140.68	0.000	0.849
**F-TIME**	0.01	1	0.01	131.73	0.000	0.840
**KG-TYPE × F-TIME**	0.00	1	0.00	45.16	0.000	0.644
**Error**	0.00	25	0.00			
**Total**	2.95	30				
**Corrected Total**	0.37	29				
***R^2** = 0.994 (**Adjusted R^2** = 0.993)						
**Zinc**						
**Corrected Model**	0.05*	4	0.01	96.82	0.000	0.939
**Intercept**	1.51	1	1.51	12829.90	0.000	0.998
**KG-TYPE**	0.00	1	0.00	4.21	0.051	0.144
**F-TIME**	0.04	1	0.04	337.96	0.000	0.931
**KG-TYPE × F-TIME**	0.00	1	0.00	5.86	0.023	0.190
**Error**	0.00	25	0.00			
**Total**	1.60	30				
**Corrected Total**	0.05	29				
***R^2** = 0.939 (**Adjusted R^2** = 0.930)						
**Copper**						
**Corrected Model**	0.00*	4	0.00	4.75	0.005	0.432
**Intercept**	1.88	1	1.88	21148.21	0.000	0.999
**KG-TYPE**	0.00	1	0.00	5.09	0.033	0.169
**F-TIME**	0.00	1	0.00	3.50	0.073	0.123
**KG-TYPE × F-TIME**	0.00	1	0.00	8.40	0.008	0.252
**Error**	0.00	25	0.00			

**Total**	1.99	30				
**Corrected Total**	0.00	29				
***R^2** = 0.432 (**Adjusted R^2** = 0.341)						
**Selenium**						
**Corrected Model**	0.06*	4	0.01	163.97	0.000	0.963
**Intercept**	1.65	1	1.65	18667.63	0.000	0.999
**KG-TYPE**	0.02	1	0.02	203.73	0.000	0.891
**F-TIME**	0.01	1	0.01	148.53	0.000	0.856
**KG-TYPE × F-TIME**	0.02	1	0.02	288.02	0.000	0.920
**Error**	0.00	25	0.00			
**Total**	1.77	30				
**Corrected Total**	0.06	29				
***R^2** = 0.963 (**Adjusted R^2** = 0.957)						

**Table S3**. Summary of the general linear model assessing the effects of kefir grain type (KG-TYPE), fermentation time (F-TIME), and their interaction (KG-TYPE × F-TIME) on volatile compound concentrations in unfermented juice and in samples fermented with milk kefir grains (MKG) or water kefir grains (WKG) for 24 or 48 h. df: degrees of freedom, ηp²: partial eta-squared, represents an estimate of the effect size for the corresponding source.						
**1-Octanol**						
**Tests of Between-Subjects Effects**						
**Source**	**Type III Sum of Squares**	**df**	**Mean Square**	**F**	**Sig.**	**ηp²**
**Corrected Model**	945.41*	4	236.35	4781.88	0.000	0.999
**Intercept**	3393.37	1	3393.37	68654.55	0.000	1.000
**KG-TYPE**	35.98	1	35.98	728.05	0.000	0.967
**F-TIME**	619.51	1	619.51	12533.97	0.000	0.998
**KG-TYPE × F-TIME**	24.06	1	24.06	486.86	0.000	0.951
**Error**	1.24	25	0.05			
**Total**	5001.78	30				
**Corrected Total**	946.65	29				
***R^2** = 0.999 (**Adjusted R^2** = 0.998)						
**1-Hexanol**						
**Corrected Model**	106.79*	4	26.70	88.39	0.000	0.934
**Intercept**	649.00	1	649.00	2148.79	0.000	0.988
**KG-TYPE**	0.40	1	0.40	1.33	0.259	0.051
**F-TIME**	3.64	1	3.64	12.05	0.002	0.325
**KG-TYPE × F-TIME**	0.01	1	0.01	0.03	0.860	0.001
**Error**	7.55	25	0.30			
**Total**	929.78	30				
**Corrected Total**	114.34	29				
***R^2** = 0.934 (**Adjusted R^2** = 0.923)						
**(Z)-3-Hexen-1-ol**						
**Corrected Model**	0.78*	4	0.19	983.28	0.000	0.994
**Intercept**	2.92	1	2.92	14699.38	0.000	0.998
**KG-TYPE**	0.08	1	0.08	413.94	0.000	0.943
**F-TIME**	0.42	1	0.42	2112.62	0.000	0.988
**KG-TYPE × F-TIME**	0.01	1	0.01	65.94	0.000	0.725
**Error**	0.00	25	0.00			
**Total**	4.30	30				
**Corrected Total**	0.78	29				
***R^2** = 0.994 (**Adjusted R^2** = 0.993)						
**Linalool**						
**Corrected Model**	118.90*	4	29.72	23.61	0.000	0.791
**Intercept**	2115.04	1	2115.04	1679.79	0.000	0.985
**KG-TYPE**	7.33	1	7.33	5.82	0.023	0.189
**F-TIME**	86.17	1	86.17	68.44	0.000	0.732

**KG-TYPE × F-TIME**	10.62	1	10.62	8.44	0.008	0.252
**Error**	31.48	25	1.26			
**Total**	2297.87	30				
**Corrected Total**	150.38	29				
***R²** = 0.791 (**Adjusted R²** = 0.757)						
**3-Methyl-1-butanol**						
**Corrected Model**	226.36*	4	56.59	1009.29	0.000	0.994
**Intercept**	423.12	1	423.12	7546.28	0.000	0.997
**KG-TYPE**	102.11	1	102.11	1821.12	0.000	0.986
**F-TIME**	53.68	1	53.68	957.30	0.000	0.975
**KG-TYPE × F-TIME**	24.56	1	24.56	438.09	0.000	0.946
**Error**	1.40	25	0.06			
**Total**	744.31	30				
**Corrected Total**	227.77	29				
***R²** = 0.994 (**Adjusted R²** = 0.993)						
**β-Citronellol**						
**Corrected Model**	11.73*	4	2.93	4707.74	0.000	0.999
**Intercept**	116.94	1	116.94	187669.29	0.000	1.000
**KG-TYPE**	1.78	1	1.78	2862.57	0.000	0.991
**F-TIME**	3.43	1	3.43	5503.52	0.000	0.995
**KG-TYPE × F-TIME**	0.42	1	0.42	670.64	0.000	0.964
**Error**	0.02	25	0.00			
**Total**	148.42	30				
**Corrected Total**	11.75	29				
***R²** = 0.999 (**Adjusted R²** = 0.998)						
**Geraniol**						
**Corrected Model**	69.66*	4	17.416	59.43	0.000	0.905
**Intercept**	279.94	1	279.94	955.20	0.000	0.974
**KG-TYPE**	4.60	1	4.60	15.71	0.001	0.386
**F-TIME**	0.57	1	0.57	1.94	0.176	0.072
**KG-TYPE × F-TIME**	27.55	1	27.55	94.00	0.000	0.790
**Error**	7.33	25	0.29			
**Total**	423.63	30				
**Corrected Total**	76.99	29				
***R²** = 0.905 (**Adjusted R²** = 0.890)						
**Terpinen-4-ol**						
**Corrected Model**	191.68*	4	47.921	82.65	0.000	0.930
**Intercept**	2706.95	1	2706.95	4668.64	0.000	0.995
**KG-TYPE**	56.83	1	56.83	98.02	0.000	0.797
**F-TIME**	57.61	1	57.61	99.36	0.000	0.799
**KG-TYPE × F-TIME**	52.45	1	52.45	90.46	0.000	0.783

**Error**	14.49	25	0.58			
**Total**	2939.14	30				
**Corrected Total**	206.18	29				
***R^2** = 0.930 (**Adjusted R^2** = 0.918)						
**α-Terpineol**						
**Corrected Model**	222.70*	4	55.67	976.83	0.000	0.994
**Intercept**	903.61	1	903.61	15854.22	0.000	0.998
**KG-TYPE**	69.23	1	69.23	1214.74	0.000	0.980
**F-TIME**	69.50	1	69.50	1219.45	0.000	0.980
**KG-TYPE × F-TIME**	56.58	1	56.58	992.79	0.000	0.975
**Error**	1.42	25	0.06			
**Total**	1103.24	30				
**Corrected Total**	224.12	29				
***R^2** = 0.994 (**Adjusted R^2** = 0.993)						
**trans-Carveol**						
**Corrected Model**	6.28*	4	1.57	162.29	0.000	0.963
**Intercept**	70.95	1	70.95	7331.19	0.000	0.997
**KG-TYPE**	0.00	1	0.00	0.54	0.468	0.021
**F-TIME**	5.34	1	5.34	551.44	0.000	0.957
**KG-TYPE × F-TIME**	0.02	1	0.02	1.66	0.210	0.062
**Error**	0.24	25	0.01			
**Total**	77.53	30				
**Corrected Total**	6.52	29				
***R^2** = 0.963 (**Adjusted R^2** = 0.957)						
**Nerol**						
**Corrected Model**	3.53*	4	0.88	224.90	0.000	0.973
**Intercept**	3.13	1	3.13	798.85	0.000	0.970
**KG-TYPE**	0.35	1	0.35	88.63	0.000	0.780
**F-TIME**	0.35	1	0.35	88.63	0.000	0.780
**KG-TYPE × F-TIME**	0.35	1	0.35	88.63	0.000	0.780
**Error**	0.10	25	0.00			
**Total**	5.72	30				
**Corrected Total**	3.63	29				
***R^2** = 0.973 (**Adjusted R^2** = 0.969)						
**2-Phenylethanol**						
**Corrected Model**	74.24*	4	18.560	1002.42	0.000	0.994
**Intercept**	138.87	1	138.87	7500.09	0.000	0.997
**KG-TYPE**	1.10	1	1.10	59.65	0.000	0.705
**F-TIME**	25.68	1	25.68	1387.23	0.000	0.982
**KG-TYPE × F-TIME**	0.39	1	0.39	21.12	0.000	0.458
**Error**	0.46	25	0.02			

**Total**	262.95	30				
**Corrected Total**	74.70	29				
***R^2** = 0.994 (**Adjusted R^2** = 0.993)						
**Decanal**						
**Tests of Between-Subjects Effects**						
**Source**	**Type III Sum of Squares**	**df**	**Mean Square**	**F**	**Sig.**	**ηp²**
**Corrected Model**	1646.76*	4	411.690	23472.38	0.000	1.000
**Intercept**	982.36	1	982.36	56009.25	0.000	1.000
**KG-TYPE**	2.36	1	2.36	134.81	0.000	0.844
**F-TIME**	2.12	1	2.12	121.02	0.000	0.829
**KG-TYPE × F-TIME**	0.71	1	0.71	40.37	0.000	0.618
**Error**	0.44	25	0.02			
**Total**	2160.92	30				
**Corrected Total**	1647.20	29				
***R^2** = 1.000 (**Adjusted R^2** = 1.000)						
**Dodecanal**						
**Corrected Model**	81.09*	4	20.27	3808.22	0.000	0.998
**Intercept**	68.92	1	68.92	12947.16	0.000	0.998
**KG-TYPE**	0.11	1	0.11	20.52	0.000	0.451
**F-TIME**	1.70	1	1.70	319.90	0.000	0.928
**KG-TYPE × F-TIME**	0.68	1	0.68	127.28	0.000	0.836
**Error**	0.13	25	0.00			
**Total**	122.73	30				
**Corrected Total**	81.22	29				
***R^2** = 0.998 (**Adjusted R^2** = 0.998)						
**Hexanal**						
**Corrected Model**	2.12*	4	0.53	1952.06	0.000	0.997
**Intercept**	7.55	1	7.55	27787.66	0.000	0.999
**KG-TYPE**	0.33	1	0.33	1205.61	0.000	0.980
**F-TIME**	0.87	1	0.87	3213.41	0.000	0.992
**KG-TYPE × F-TIME**	0.89	1	0.89	3286.12	0.000	0.992
**Error**	0.01	25	0.00			
**Total**	10.32	30				
**Corrected Total**	2.13	29				
***R^2** = 0.997 (**Adjusted R^2** = 0.996)						
**Neral**						
**Corrected Model**	10.44*	4	2,61	6607.44	0.000	0.999
**Intercept**	7.86	1	7.86	19916.27	0.000	0.999
**KG-TYPE**	0.04	1	0.04	99.57	0.000	0.799
**F-TIME**	0.23	1	0.23	593.95	0.000	0.960

**KG-TYPE × F-TIME**	0.04	1	0.04	99.57	0.000	0.799
**Error**	0.01	25	0.00			
**Total**	14.99	30				
**Corrected Total**	10.45	29				
***R^2** = 0.999 (**Adjusted R^2** = 0.999)						
**Geranial**						
**Corrected Model**	36.51*	4	9.13	9121.79	0.000	00.999
**Intercept**	27.83	1	27.83	27814.26	0.000	0.999
**KG-TYPE**	0.19	1	0.19	186.31	0.000	0.882
**F-TIME**	0.89	1	0.89	888.50	0.000	0.973
**KG-TYPE × F-TIME**	0.19	1	0.19	186.31	0.000	0.882
**Error**	0.02	25	0.00			
**Total**	52.72	30				
**Corrected Total**	36.54	29				
***R^2** = 0.999 (**Adjusted R^2** = 0.999)						
**Octanoic acid**						
**Tests of Between-Subjects Effects**						
**Source**	**Type III Sum of Squares**	**df**	**Mean Square**	**F**	**Sig.**	**ηp^2**
**Corrected Model**	0.32*	4	0.08	1132.21	0.000	0.995
**Intercept**	0.06	1	0.06	835.24	0.000	0.971
**KG-TYPE**	0.10	1	0.10	1415.26	0.000	0.983
**F-TIME**	0.10	1	0.10	1415.26	0.000	0.983
**KG-TYPE × F-TIME**	0.10	1	0.10	1415.26	0.000	0.983
**Error**	0.00	25	0.00			
**Total**	0.40	30				
**Corrected Total**	0.32	29				
***R^2** = 0.995 (**Adjusted R^2** = 0.994)						
**Dodecanoic acid**						
**Corrected Model**	0.25*	4	0.06	3156.55	0.000	0.998
**Intercept**	0.05	1	0.05	2328.60	0.000	0.989
**KG-TYPE**	0.08	1	0.08	3945.69	0.000	0.994
**F-TIME**	0.08	1	0.08	3945.69	0.000	0.994
**KG-TYPE × F-TIME**	0.08	1	0.08	3945.69	0.000	0.994
**Error**	0.00	25	0.00			
**Total**	0.32	30				
**Corrected Total**	0.25	29				
***R^2** = 0.998 (**Adjusted R^2** = 0.998)						
**n-Decanoic acid**						
**Corrected Model**	62.29*	4	15.57	16502.62	0.000	1.000
**Intercept**	14.07	1	14.07	14914.85	0.000	0.998

**KG-TYPE**	23.85	1	23.85	25272.39	0.000	0.999
**F-TIME**	16.84	1	16.84	17841.81	0.000	0.999
**KG-TYPE × F-TIME**	16.84	1	16.84	17841.81	0.000	0.999
**Error**	0.02	25	0.00			
**Total**	81.39	30				
**Corrected Total**	62.31	29				
***R^2** = 1.000 (**Adjusted R^2** = 1.000)						
**Ethyl butanoate**						
**Tests of Between-Subjects Effects**						
**Source**	**Type III Sum of Squares**	**df**	**Mean Square**	**F**	**Sig.**	**ηp²**
**Corrected Model**	552.24*	4	138.06	205.26	0.000	0.970
**Intercept**	380.89	1	380.89	566.29	0.000	0.958
**KG-TYPE**	7.30	1	7.30	10.86	0.003	0.303
**F-TIME**	426.06	1	426.06	633.45	0.000	0.962
**KG-TYPE × F-TIME**	0.39	1	0.39	0.58	0.452	0.023
**Error**	16.81	25	0.67			
**Total**	1080.29	30				
**Corrected Total**	569.05	29				
***R^2** = 0.970 (**Adjusted R^2** = 0.966)						
**Ethyl hexanoate**						
**Corrected Model**	26.61*	4	6.65	1013.99	0.000	0.994
**Intercept**	56.39	1	56.39	8595.88	0.000	0.997
**KG-TYPE**	0.82	1	0.82	125.66	0.000	0.834
**F-TIME**	6.84	1	6.84	1042.06	0.000	0.977
**KG-TYPE × F-TIME**	3.53	1	3.53	537.64	0.000	0.956
**Error**	0.16	25	0.01			
**Total**	101.39	30				
**Corrected Total**	26.77	29				
***R^2** = 0.994 (**Adjusted R^2** = 0.993)						
**Ethyl octanoate**						
**Corrected Model**	5.01*	4	1.25	3449.54	0.000	0.998
**Intercept**	11.92	1	11.92	32803.96	0.000	0.999
**KG-TYPE**	0.58	1	0.58	1601.49	0.000	0.985
**F-TIME**	0.38	1	0.38	1041.65	0.000	0.977
**KG-TYPE × F-TIME**	0.01	1	0.01	38.11	0.000	0.604
**Error**	0.01	25	0.00			
**Total**	21.18	30				
**Corrected Total**	5.02	29				
***R^2** = 0.998 (**Adjusted R^2** = 0.998)						
**Octyl acetate**						

**Corrected Model**	10.44*	4	2,61	6607.44	0.000	0.999
**Intercept**	7.86	1	7.86	19916.27	0.000	0.999
**KG-TYPE**	0.04	1	0.04	99.57	0.000	0.799
**F-TIME**	0.23	1	0.23	593.95	0.000	0.960
**KG-TYPE × F-TIME**	0.04	1	0.04	99.57	0.000	0.799
**Error**	0.01	25	0.00			
**Total**	14.99	30				
**Corrected Total**	10.45	29				
***R^2** = 0.999 (**Adjusted R^2** = 0.999)						
**3-Octanone**						
**Tests of Between-Subjects Effects**						
**Source**	**Type III Sum of Squares**	**df**	**Mean Square**	**F**	**Sig.**	**ηp²**
**Corrected Model**	0.46*	4	0.11	1037.29	0.000	0.994
**Intercept**	0.92	1	0.92	8290.29	0.000	0.997
**KG-TYPE**	0.04	1	0.04	357.95	0.000	0.935
**F-TIME**	0.10	1	0.10	893.69	0.000	0.973
**KG-TYPE × F-TIME**	0.01	1	0.01	88.01	0.000	0.779
**Error**	0.00	25	0.00			
**Total**	1.71	30				
**Corrected Total**	0.46	29				
***R^2** = 0.994 (**Adjusted R^2** = 0.993)						
**δ-Carvone**						
**Corrected Model**	0.11*	4	0.03	26.69	0.000	0.810
**Intercept**	2.61	1	2.61	2517.35	0.000	0.990
**KG-TYPE**	0.03	1	0.03	25.62	0.000	0.506
**F-TIME**	0.04	1	0.04	42.23	0.000	0.628
**KG-TYPE × F-TIME**	0.00	1	0.00	4.84	0.037	0.162
**Error**	0.03	25	0.00			
**Total**	3.04	30				
**Corrected Total**	0.14	29				
***R^2** = 0.810 (**Adjusted R^2** = 0.780)						
**Nootkatone**						
**Corrected Model**	4.84*	4	1.21	233.29	0.000	0.974
**Intercept**	26.64	1	26.64	5133.84	0.000	0.995
**KG-TYPE**	0.20	1	0.20	37.82	0.000	0.602
**F-TIME**	0.87	1	0.87	167.91	0.000	0.870
**KG-TYPE × F-TIME**	2.62	1	2.62	505.83	0.000	0.953
**Error**	0.13	25	0.00			
**Total**	35.82	30				
**Corrected Total**	4.97	29				

***R**^{2} = 0.974 (**Adjusted R**^{2} = 0.970)						
**D-Limonene**						
**Tests of Between-Subjects Effects**						
**Source**	**Type III Sum of Squares**	**df**	**Mean Square**	**F**	**Sig.**	**ηp²**
**Corrected Model**	49098.52*	4	12274.63	3676.29	0.000	0.998
**Intercept**	156951.06	1	156951.06	47007.28	0.000	0.999
**KG-TYPE**	606.29	1	606.29	181.59	0.000	0.879
**F-TIME**	5436.49	1	5436.49	1628.24	0.000	0.985
**KG-TYPE × F-TIME**	5325.41	1	5325.41	1594.97	0.000	0.985
**Error**	83.47	25	3.34			
**Total**	179708.98	30				
**Corrected Total**	49181.99	29				
***R**^{2} = 0.998 (**Adjusted R**^{2} = 0.998)						
**β-Myrcene**						
**Corrected Model**	1473.72*	4	368.43	754.03	0.000	0.992
**Intercept**	1987.41	1	1987.41	4067.44	0.000	0.994
**KG-TYPE**	163.81	1	163.81	335.26	0.000	0.931
**F-TIME**	359.90	1	359.90	736.57	0.000	0.967
**KG-TYPE × F-TIME**	347.96	1	347.96	712.14	0.000	0.966
**Error**	12.21	25	.489			
**Total**	3087.89	30				
**Corrected Total**	1485.93	29				
***R**^{2} = 0.992 (**Adjusted R**^{2} = 0.990)						
**α-Pinene**						
**Corrected Model**	22.89*	4	5.72	2734.04	0.000	0.998
**Intercept**	23.82	1	23.82	11380.66	0.000	0.998
**KG-TYPE**	5.75	1	5.75	2746.50	0.000	0.991
**F-TIME**	3.54	1	3.54	1689.37	0.000	0.985
**KG-TYPE × F-TIME**	3.54	1	3.54	1689.37	0.000	0.985
**Error**	0.05	25	0.00			
**Total**	41.15	30				
**Corrected Total**	22.94	29				
***R**^{2} = 0.998 (**Adjusted R**^{2} = 0.997)						
**β-Ocimene**						
**Corrected Model**	2.88*	4	0.72	164.51	0.000	0.963
**Intercept**	15.99	1	15.99	3650.23	0.000	0.993
**KG-TYPE**	0.03	1	0.03	6.95	0.014	0.217
**F-TIME**	0.01	1	0.01	3.11	0.090	0.111
**KG-TYPE × F-TIME**	0.03	1	0.03	6.99	0.014	0.219
**Error**	0.11	25	0.00			

**Total**	16.78	30				
**Corrected Total**	2.99	29				
***R²** = 0.963 (**Adjusted R²** = 0.958)						
**γ-Terpinene**						
**Corrected Model**	44.76*	4	11.19	1229.09	0.000	0.995
**Intercept**	83.10	1	83.10	9127.98	0.000	0.997
**KG-TYPE**	0.00	1	0.00	0.29	0.594	0.012
**F-TIME**	0.16	1	0.16	17.12	0.000	0.406
**KG-TYPE × F-TIME**	0.01	1	0.01	0.64	0.430	0.025
**Error**	0.23	25	.009			
**Total**	105.72	30				
**Corrected Total**	44.98	29				
***R²** = 0.995 (**Adjusted R²** = 0.994)						
**α-Copaene**						
**Corrected Model**	2.71*	4	0.68	687.97	0.000	0.991
**Intercept**	3.60	1	3.60	3659.40	0.000	0.993
**KG-TYPE**	0.10	1	0.10	98.81	0.000	0.798
**F-TIME**	0.09	1	0.09	92.15	0.000	0.787
**KG-TYPE × F-TIME**	0.01	1	0.01	7.92	0.009	0.241
**Error**	0.02	25	0.00			
**Total**	5.22	30				
**Corrected Total**	2.73	29				
***R²** = 0.991 (**Adjusted R²** = 0.990)						
**Caryophyllene**						
**Corrected Model**	12.98*	4	3.25	88211.79	0.000	1.000
**Intercept**	18.35	1	18.35	498695.26	0.000	1.000
**KG-TYPE**	2.25	1	2.25	61240.61	0.000	1.000
**F-TIME**	0.53	1	0.53	14328.53	0.000	0.998
**KG-TYPE × F-TIME**	0.14	1	0.14	3866.06	0.000	0.994
**Error**	0.00	25	0.00			
**Total**	26.34	30				
**Corrected Total**	12.98	29				
***R²** = 1.000 (**Adjusted R²** = 1.000)						
**1,3,8-p-Menthatriene**						
**Corrected Model**	1.44*	4	0.36	233.30	0.000	0.974
**Intercept**	8.80	1	8.80	5712.77	0.000	0.996
**KG-TYPE**	0.45	1	0.45	292.11	0.000	0.921
**F-TIME**	0.32	1	0.32	204.93	0.000	0.891
**KG-TYPE × F-TIME**	0.35	1	0.35	225.33	0.000	0.900
**Error**	0.04	25	0.00			
**Total**	9.96	30				

**Corrected Total**	1.47	29				
***R^2** = 0.974 (**Adjusted R^2** = 0.970)						
**α-Humulene**						
**Corrected Model**	0.44*	4	0.11	5786.76	0.000	0.999
**Intercept**	0.34	1	0.34	17997.54	0.000	0.999
**KG-TYPE**	0.02	1	0.02	823.85	0.000	0.971
**F-TIME**	0.02	1	0.02	823.85	0.000	0.971
**KG-TYPE × F-TIME**	0.02	1	0.02	823.85	0.000	0.971
**Error**	0.00	25	0.00			
**Total**	0.64	30				
**Corrected Total**	0.44	29				
***R^2** = 0.999 (**Adjusted R^2** = 0.999)						
**Sabinene**						
**Corrected Model**	2.28*	4	0.57	529.42	0.000	0.988
**Intercept**	19.44	1	19.44	18048.23	0.000	0.999
**KG-TYPE**	0.04	1	0.04	37.03	0.000	0.597
**F-TIME**	0.07	1	0.07	69.27	0.000	0.735
**KG-TYPE × F-TIME**	0.02	1	0.02	15.31	0.001	0.380
**Error**	0.03	25	0.00			
**Total**	19.82	30				
**Corrected Total**	2.31	29				
***R^2** = 0.988 (**Adjusted R^2** = 0.986)						

Table S4. Classification of volatile organic compounds (VOCs) into aroma categories and the corresponding sums of odor activity values (ΣOAVs).

Aroma category	VOCs assigned to each aroma category	ΣOAV (UOJ)	ΣOAV (OJMKG24h)	ΣOAV (OJMKG48h)	ΣOAV (OJWKG24h)	ΣOAV (OJWKG48h)
Citrus	Geraniol, decanal, neral, geranial, ethyl hexanoate, D-limonene, γ-terpinene.	7619.78	1789.62	2498.17	3215.97	1957.53
Floral	Linalool, β-citronellol, terpinen-4-ol, 2-phenylethanol, dodecanal, octyl acetate, α-humulene, farnesene.	5176.80	2751.50	3965.58	4829.57	3935.48
Fruity/sweet	3-Methyl-1-butanol, ethyl butanoate, ethyl octanoate, 3-octanone.	275.81	2087.07	11478.74	469.08	10165.77
Green/fresh/herbaceous	1-Octanol, 1-hexanol, (Z)-3-hexen-1-ol, trans-carveol, hexanal, nootkatone, β-ocimene, sabinene.	458.95	1220.67	1574.80	1159.01	1404.45
Balsamic	β-Myrcene.	13555.75	12800.24	0.00	2099.81	1991.88
Woody/Spicy	α-Pinene, α-copaene, caryophyllene.	311.38	153.48	15.08	55.92	34.40
Minty/Camphoraceous	α-Terpineol, δ-carvone, 1,3,8-p-menthatriene.	56.94	48.96	43.18	14.36	46.11
Total ∑OAVs		27455.41	20851.54	19575.55	11843.72	19535.62

*VOCs were assigned to aroma categories according to their dominant aroma descriptors. Each compound was assigned exclusively to a single aroma category, following the approach commonly used in aroma-series analysis to avoid double counting and preserve the total ΣOAV.
